# Supplementary material for: Mental and somatic disorders and the subsequent risk of all-cause and cause-specific mortality in refugees, non-refugee migrants and the Swedish-born youth: a population-based cohort study in Sweden
Source: BMJ Open. 2022 May 11;12(5):e054351. doi: 10.1136/bmjopen-2021-054351 (PMC9096569; doi:10.1136/bmjopen-2021-054351)
Supplement: Supplementary data [file bmjopen-2021-054351supp001.pdf]

Supplementary file showing the directed acyclic graphs (DAG)

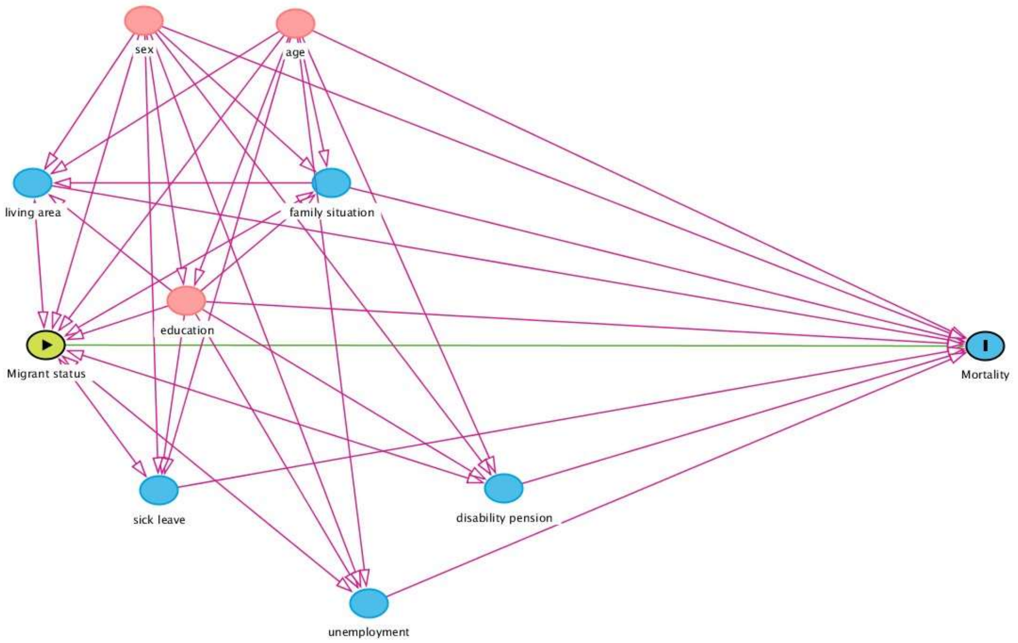

DAG for exposure (migrant status) and outcome (mortality) **unadjusted** for sociodemographic and Labour market marginalisation factors

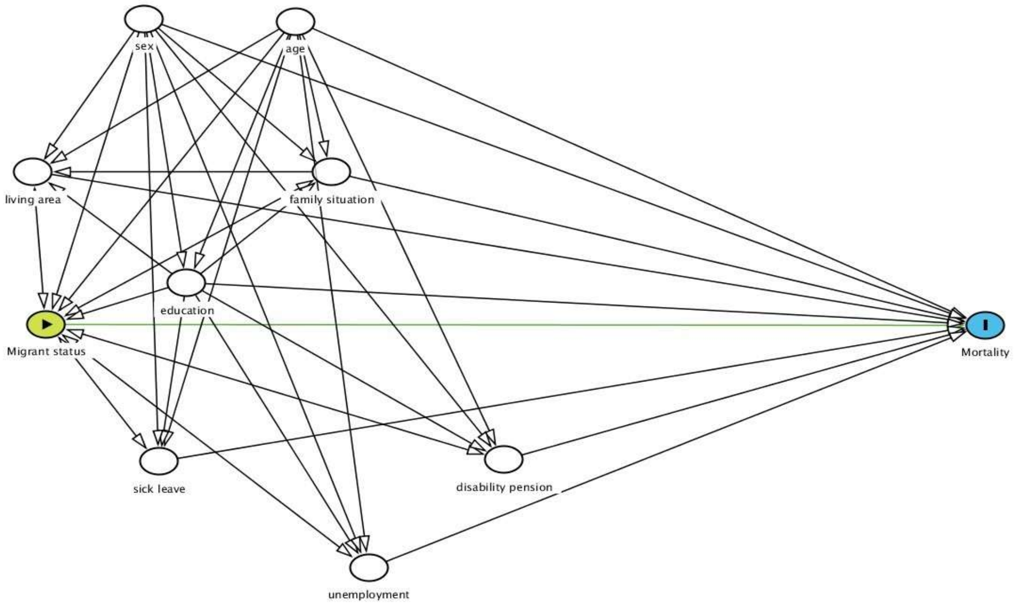

DAG for exposure (migrant status) and outcome (mortality) **adjusted** for **1)** sociodemographic and **2)** labour market marginalisation (LMM) factors

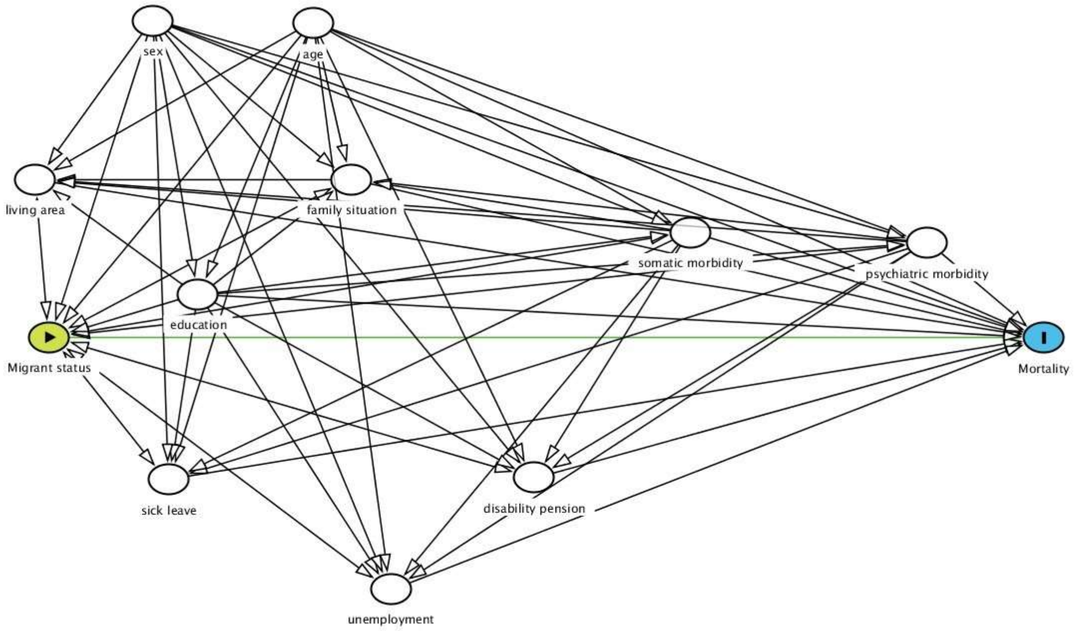

DAG for exposure (migrant status) and outcome (mortality) **adjusted** for **1)** sociodemographic, **2)** LMM and **3)** morbidity factors
