# Supplementary material for: Mental and somatic disorders and the subsequent risk of all-cause and cause-specific mortality in refugees, non-refugee migrants and the Swedish-born youth: a population-based cohort study in Sweden
Source: BMJ Open. 2022 May 11;12(5):e054351. doi: 10.1136/bmjopen-2021-054351 (PMC9096569; doi:10.1136/bmjopen-2021-054351)
Supplement: Author's manuscript [file bmjopen-2021-054351.draft_revisions.pdf]

BMJ Open is committed to open peer review. As part of this commitment we make the peer review history of every article we publish publicly available.

When an article is published we post the peer reviewers' comments and the authors' responses online. We also post the versions of the paper that were used during peer review. These are the versions that the peer review comments apply to.

The versions of the paper that follow are the versions that were submitted during the peer review process. They are not the versions of record or the final published versions. They should not be cited or distributed as the published version of this manuscript.

BMJ Open is an open access journal and the full, final, typeset and author-corrected version of record of the manuscript is available on our site with no access controls, subscription charges or pay-per-view fees (http://bmjopen.bmj.com).

If you have any questions on BMJ Open's open peer review process please email info.bmjopen@bmj.com

BMJ Open

Mental and somatic disorders and the subsequent risk of all-cause and cause-specific mortality in young adults – are there differences between refugees, non-refugee migrants and individuals born in Sweden?

Journal:	*BMJ Open*
Manuscript ID	bmjopen-2021-054351
Article Type:	Original research
Date Submitted by the Author:	09-Jun-2021

Complete List of Authors:	Helgesson, Magnus; Karolinska Institutet, Department of Clinical Neuroscience<br>Björkenstam, Emma; Karolinska Institutet, Department of Clinical Neuroscience; Uppsala University, Department of Neuroscience<br>Filatova, Svetlana; Karolinska Institutet, Department of Clinical Neuroscience<br>Rahman, Syed; Karolinska Institutet, Department of Clinical Neuroscience<br>Cullen, Alexis; Karolinska Institutet, Department of Clinical Neuroscience; King's College London, Department of Psychosis Studies<br>Dorner, Thomas; Karolinska Institutet, Department of Clinical Neuroscience; Karl-Landsteiner Institute for Health Promotion Research<br>Gémes, Katalin; Karolinska Institutet, Department of Clinical Neuroscience<br>Mittendorfer-Rutz, Ellenor; Karolinska Institutet, Department of Clinical Neuroscience

Keywords:	EPIDEMIOLOGY, Suicide & self-harm < PSYCHIATRY, PUBLIC HEALTH

SCHOLARONE™
Manuscripts

I, the Submitting Author has the right to grant and does grant on behalf of all authors of the Work (as defined in the below author licence), an exclusive licence and/or a non-exclusive licence for contributions from authors who are: i) UK Crown employees; ii) where BMJ has agreed a CC-BY licence shall apply, and/or iii) in accordance with the terms applicable for US Federal Government officers or employees acting as part of their official duties; on a worldwide, perpetual, irrevocable, royalty-free basis to BMJ Publishing Group Ltd ("BMJ") its licensees and where the relevant Journal is co-owned by BMJ to the co-owners of the Journal, to publish the Work in this journal and any other BMJ products and to exploit all rights, as set out in our licence.

The Submitting Author accepts and understands that any supply made under these terms is made by BMJ to the Submitting Author unless you are acting as an employee on behalf of your employer or a postgraduate student of an affiliated institution which is paying any applicable article publishing charge ("APC") for Open Access articles. Where the Submitting Author wishes to make the Work available on an Open Access basis (and intends to pay the relevant APC), the terms of reuse of such Open Access shall be governed by a Creative Commons licence – details of these licences and which Creative Commons licence will apply to this Work are set out in our licence referred to above.

Other than as permitted in any relevant BMJ Author's Self Archiving Policies, I confirm this Work has not been accepted for publication elsewhere, is not being considered for publication elsewhere and does not duplicate material already published. I confirm all authors consent to publication of this Work and authorise the granting of this licence.

For peer review only

Mental and somatic disorders and the subsequent risk of all-cause and cause-specific mortality in young adults – are there differences between refugees, non-refugee migrants and individuals born in Sweden?

Magnus Helgesson[1], Emma Björkenstam[1,2], Svetlana Filatova[1], Syed Rahman[1], Alexis E. Cullen [1,3], Thomas E. Dorner [1,4], Katalin Gemes[1], Ellenor Mittendorfer-Rutz[1*]

1 Division of Insurance Medicine, Department of Clinical Neuroscience, Karolinska Institutet, SE-171 77 Stockholm, Sweden

2 Department of Neuroscience, Uppsala University, Uppsala, Sweden

3 Department of Psychosis Studies, Institute of Psychiatry, Psychology & Neuroscience, King's College London, UK

4 Karl-Landsteiner Institute for Health Promotion Research, 3454 Sitzenberg-Reidling, Austria.

Corresponding author:

Ellenor Mittendorfer-Rutz, Professor, PhD

Ellenor.mittendorfer-rutz@ki.se

Karolinska Institutet

Word count: 2931

For peer review only - http://bmjopen.bmj.com/site/about/guidelines.xhtml

Abstract

Introduction: The aims were to investigate the associations between specific mental and somatic disorders and subsequent all-cause and cause-specific mortality (suicide and external causes) in young refugees and non-refugee migrants compared to Swedish-born individuals of similar age. **Methods:** The study population of this register study comprised all 1,003,760 individuals (including 40,305 refugees and 31,687 non-refugee migrants), 16-25 years old, residing in Sweden on 31 December 2004. Crude and multivariate Hazard ratios (HR) with 95% Confidence intervals (CI) were yielded by performing Cox regression models with a follow-up from 2005 to 2016. **Results:** Refugees and non-refugee migrants had about a 20 percent lower risk of all-cause mortality and mortality due to external causes compared to Swedish-born individuals, and an even greater reduction in suicide risk (HR 0.51 and 0.63 for non-refugees and refugees, respectively). When restricted to those with a mental or somatic disorder, a lower risk of both general and specific mortality was also found among both refugees and non-refugee migrants compared to Swedish-born individuals. Refugees had, however, equal point estimates of all-cause mortality associated with substance misuse disorder and neoplasms as their Swedish-born peers with these disorders. **Conclusions:** With few exceptions, young migrants with specific mental and somatic disorders have a mortality advantage compared to their Swedish-born peers with the same disorders. Further research on protective factors is warranted.

For peer review only

Strengths and limitations of this study

- This study builds on a longitudinal population-based design and used national registers with high completeness and validity.
- The large cohort size allowed for detailed analyses of different types of mental and somatic disorders, the ability to adjust for important confounders and a rare outcome.
- Information on exposure relied solely on register data from specialised care, and thus we have likely captured the most severe cases.
- Potential differences in healthcare-seeking behaviour in Swedish-born individuals and migrants may affect our findings.
- Further, the rare outcome may lead to lower statistical power for some of the analyses.

For peer review only

For peer review only - http://bmjopen.bmj.com/site/about/guidelines.xhtml

What is already known?

Studies investigating mortality across all age groups have reported a lower mortality risk in refugees compared to the host population.

What are the new findings?

Refugees and non-refugee migrants had about a 20 percent lower risk of all-cause mortality and mortality due to external causes compared to Swedish-born individuals, and an even greater reduction in suicide risk.

When restricted to those with a mental or somatic disorder, a lower risk of both general and specific mortality was also found among both refugees and non-refugee migrants compared to Swedish-born individuals.

Refugees had, however, equal point estimates of all-cause mortality associated with substance misuse disorder and neoplasms as their Swedish-born peers with these disorders.

What do the new findings imply?

Further research on protective factors is warranted.

For peer review only

For peer review only - http://bmjopen.bmj.com/site/about/guidelines.xhtml

Introduction

During the past few years, there has been a substantial increase in global migration. In year 2017, there were 258 million migrants worldwide, including 25.4 million refugees.[1 2] Sweden is one of the largest European recipients of refugees, during the last two decades, more than three quarters of a million refugees sought asylum in Sweden.[3] About one third of them were children, and a large proportion arrived in Sweden unaccompanied by parents or relatives. This development has the potential to be an eminent public health challenge, as many of the young refugees have experienced traumatic events, such as bereavement and violence, during war.[3-5] For these reasons, studies report a higherrisk for mental disorders, particularly post-traumatic stress disorders in young refugees compared to the host population.[6]

Despite the large numbers of young refugees worldwide and their higher morbidity risk, there is a scarcity of studies focussing on the most prominent health outcomes, i.e. mortality, in this group. Mortality is a relatively rare event among young persons in general and death are mainly attributable to external causes including suicide, injuries, accidents, and poisoning.[7-9] Studies investigating mortality across all age groups have reported a lower mortality risk in refugees compared to the host population.[10 11] This migrant mortality advantage has puzzled researchers as it occurs against a background of higher morbidity levels in these populations.[12] Explanatory models include not only in- and out-migration health selection, but also cultural features such as better health behaviour but also data artefacts.[10 12 13] There seems to be an age variation in this migrant mortality advantage, however, where protection from excess mortality does not appear to apply to children as it does among their older counterparts.[12] Young refugees might be at a particular mortality risk, in part, as their main causes of death, suicide and external causes, are strongly related to mental disorders, which are known to have an early age of onset and high prevalence rates in young refugees.[14-16] It is therefore crucial to investigate the mortality risk in young refugees.

For peer review only - http://bmjopen.bmj.com/site/about/guidelines.xhtml

Besides these higher prevalence rates of mental disorders, the occurrence of some somatic disorders, including for example, infectious diseases as well as diseases of the respiratory system and the nervous system, appear to be prevalent in refugees.[11 17] Variation in the importance of specific mental disorders between adult refugees and Swedish-born regarding the subsequent risk of suicide mortality has also been recently reported.[9] Differences of the relative importance of specific mental and somatic disorders for subsequent mortality risk in young refugees compared to young individuals in the host population are likely, but are lacking to date. Any such differences might arise from unmet healthcare and treatment needs of young refugees with specific disorders.[18] Here, adequate care in young refugees might be hampered by language barriers, differences in the clinical manifestation and the course of the underlying disease, as well as the lack of competence in transcultural medicine among health care staff in the host country.

There seem to be differences in the prevalence of both mental and somatic disorders between subgroups of migrants. A study from Sweden found that the risk of a mental disorder is higher in refugee migrants compared to non-refugee migrants.[19] Differences in the occurrence of multimorbidity between refugees, non-refugee migrants and the host population were also reported by a study from Norway.[20] Refugees have a higher risk of having experienced traumatic events, which might put them at a higher risk of developing different disorders and subsequently premature mortality compared to other migrants.[21] On the other hand, refugees and non-refugee migrants from the same countries of birth might share common culturally determined features, including the perception of health care, acceptance of treatment and health behaviour. To be able to create tailor-made interventions, there is therefore a need to study different subgroups of migrants separately.

This study aims to address the mentioned literature gaps by investigating if the risk of subsequent all-cause and cause-specific mortality differs in young refugees and non-refugee

For peer review only - http://bmjopen.bmj.com/site/about/guidelines.xhtml

migrants compared to individuals in the host population of the same age in general and related to specific mental and somatic disorders.

Methods

Study population

The study population was defined as all individuals aged 16–25 years, residing in Sweden on 31 December 2004, i.e., born between 1979 and 1988 (n = 1,081,412). Only those with complete information on their reason for settlement in Sweden were included (n = 1,048,762). For the non-refugee migrants, we excluded those who did not come from the same countries as the refugees (n = 9,008). A futher 35,994 individuals were excluded because they did not reside in Sweden during the period 2000-2004, i.e., the period during which mental and somatic disorders were measured. The final study population comprised of 1,003,760 young individuals 16-25 years, including 40,305 refugees and 31,687 non-refugee migrants.

Registers

We used the unique (de-identified) Swedish personal identity number to link information from several population-based registers. The Longitudinal Integration Database for Health Insurance and Labour Market Studies (LISA) contains data on sociodemographic and labour market factors. The Longitudinal Database for Integration Studies (STATIV) holds migration-related information, including reasons for settlement (e.g., refugee status). The National Patient Register (NPR) includes information on inpatient healthcare since 1987, and for specialised out-patient care since 2001. Diagnoses in NPR are coded according to the International Classification of Diseases, version 10 (ICD-10). The Cause of Death Register comprises information on causes and date of deaths of Swedish residents since 1952.

For peer review only - http://bmjopen.bmj.com/site/about/guidelines.xhtml

Measures

Mental disorders

Mental disorders were defined as having a main diagnosis from inpatient or specialised outpatient healthcare, as recorded in the NPR between 2000 and 2004, of the following disorders: substance misuse disorders (ICD-10: F10–19), schizophrenia/non-affective psychotic disorders (F20–29), depressive disorders (ICD-10: F32–34), anxiety disorders (ICD-10: F40–42), stress-related disorders (F43) and behavioural disorders (ICD-10: F50–59, F90–99).

Somatic disorders

Main diagnoses in specialised healthcare (inpatient and specialised outpatient healthcare 2000-2004) for somatic disorders were divided into dichotomised variables of following groups of diagnoses, recorded according to the International Classification of Disorders version 10 (ICD-10): 1) infectious and parasitic diseases (ICD-10: A00-B99), 2) neoplasm (ICD-10: C00-D48), 3) diseases of the circulatory system (ICD-10: I00-I99), 4) diseases of the respiratory system (ICD-10: J00-J99), 5) diseases of the digestive system (ICD-10: K00-K93), 6) musculoskeletal diseases (ICD-10: M00-M99), 7) diseases of the nervous system (ICD-10: G00-G99) and 8) other somatic disorders (ICD-10: E00-E09, E15-E90, H01-H99, L00-L99, N00-T99).

Outcome measures

The outcome measures were defined as both all-cause and cause-specific mortality. More specifically, we studied external causes (ICD-10: V00-Y99) as one outcome, and within this group, suicide (ICD-10: X60-84, Y10-34) was examined separately..

For peer review only - http://bmjopen.bmj.com/site/about/guidelines.xhtml

Covariates

The covariates included in the analyses were 1) socio-demographic factors: sex, age, educational level, type of living area and family situation (all variables were measured at baseline, i.e., 31st December 2004); 2) work-related factors: unemployment (0, 1-180, >180 days); sickness absence (0, 1-90, >90 days) and disability pension, all measured during 2004. Missing values in any covariates were treated as a separate category in the adjusted analyses. Categorisations of covariates are shown in Table 1.

Statistical analyses

Statistical analyses were conducted using SAS, v.9.4. Crude and multivariate analyses were performed using Cox regression models of time to diagnosis-specific and all-cause mortality during the follow-up. We assessed person-years at risk by totalling the years that the individuals were living in Sweden during the follow-up period. The entry date was defined as January 1st, 2005, and the exit date as the date of the first outcome, date of emigration, or the end of follow-up on December 31st, 2016. First, risk estimates for refugees and non-refugee migrants were compared to those for Swedish-born regarding subsequent all-cause mortality and cause-specific mortality. The association of mental and somatic disorders and subsequent all-cause mortality with refugee status was then assessed. Here, analyses were performed for any mental or somatic disorders and then we conducted separate analyses for each diagnostic group (only specific disorders where number of cases exceeded ten either in refugees or non-refugee migrants are shown in tables). We examined the associations between migrant status and each outcome in one crude and one adjusted regression model. Model 2 was adjusted for age, sex, education, family situation, type of living area, unemployment, sickness absence and disability pension, and finally morbidity (i.e., mental and somatic disorders as described above) at baseline. Analyses investigating the association with mental disorders and subsequent mortality

For peer review only - http://bmjopen.bmj.com/site/about/guidelines.xhtml

were adjusted for somatic comorbidity and vice versa. Results are presented as crude and adjusted hazard ratios (HRs and aHRs) with 95% confidence intervals (CIs).

Patient and Public Involvement statement

This is a register study building on administrative registers including approximately 1 million individuals. It was thus not appropriate or possible to involve patients or the public in the design, or conduct, or reporting, or dissemination plans of our research.

For peer review only

Results

Among refugees, a slightly higher percentage of men (53%) were found compared to both Swedish-born (51%) and non-refugee migrants (49%, Table 1). There was a higher percentage of Swedish-born individuals (17%) with college/university education compared to both refugees (12%) and non-refugee migrants (10%). Among the refugees, the majority came from Former Yugoslavian countries (44%), whereas non-refugee migrants primarily emigrated from Asian countries. Most of the migrants had lived in Sweden more than 10 years (47-57%). The percentage of of individuals who were unemployed >180 days was more than double among migrants (2.3-2.9%) compared to Swedish-born (1.4%), yet there were no differences between the groups regarding the prevalence of sickness absence and granting of disability pension. The prevalence of any specialised health care due to mental disorder was highest among non-refugee migrants (5.5%) and lowest among refugees (4.5%). Likewise, specialised heatlh care use due to a somatic disorder occurred most frequently in non-refugee migrants (54.5%) followed by refugees (53.5%) and Swedish-born individuals (51.2%).

Table 1. *Cohort and health-related characteristics, by refugee status, in individuals aged 16-25 years old residing in Sweden in 2000-2004.*

For peer review only - http://bmjopen.bmj.com/site/about/guidelines.xhtml

	All	Swedish-born individuals	Refugees	Non-refugee migrants
All, (n, row percent)	1,003,760 (100)	931,768 (93)	40,305 (4)	31,687 (3)
Sociodemographic factors at baseline				
Sex				
Women	487,254 (49)	452,142 (49)	19,041 (47)	16,071 (51)
Men	516,506 (51)	479,626 (51)	21,264 (53)	15,616 (49)
Age group				
16-20	525,724 (52)	487,676 (52)	21,378 (53)	16,670 (53)
21-25	478,036 (48)	444,092 (48)	18,927 (47)	15,017 (47)
Mean age (years, SD)	20.3 (2.9)	20.3 (2.9)	20.3 (2.8)	20.4 (2.9)
Education (years)				
Compulsory school (<9)	408,595 (41)	374,906 (40)	18,354 (46)	15,335 (48)
High school (10–12)	407,911 (41)	383,312 (41)	14,848 (37)	9,751 (31)
College or university (>12)	162,977 (16)	154,907 (17)	4,994 (12)	3,076 (10)
Missing	24,277 (2)	18,643 (2)	2,109 (5)	3,525 (11)
Family situation				
Married/living with partner without children[a]	8,915 (1)	6,342 (1)	1,518 (4)	1,055 (3)
Married/living with partner with children[a]	33,653 (3)	28,629 (3)	2,371 (6)	2,653 (8)
Single/divorced/separated/widowed without children[a]	483,651 (48)	453,573 (49)	17,067 (42)	13,011 (41)
Single/divorced/separated/widowed with children[a]	9,694 (1)	8,437 (1)	608 (2)	649 (2)
Children (≤20 years old)[2]	467,847 (47)	434,787 (47)	18,741 (46)	14,319 (45)
Type of living area				
Big city area	349,066 (35)	313,513 (34)	17,908 (44)	17,645 (56)
Intermediate (>90,000 inhabitants)	381,280 (38)	355,443 (38)	16,121 (40)	9,716 (31)
Small (rural municipalities)	273,414 (27)	262,812 (28)	6,276 (16)	4,326 (14))
Country of birth				
Horn of Africa[b]	4,675 (0)	-	1,917 (5)	2,758 (9)
Afghanistan	1,124 (0)	-	329 (1)	795 (3)
Asia (except Afghanistan, Iraq, Iran, and Syria	12,645 (1)	-	4,631 (11)	8,014 (25)
Iraq	8,464 (1)	-	4,120 (10)	4,344 (14)
Iran	7,366 (1)	-	4,773 (12)	2,593 (8)
Former Yugoslavian countries	19,576 (2)	-	17,852 (44)	1,724 (5)
Syria	1,942 (0)	-	1,423 (4)	519 (2)
Others	16,200 (2)	-	5,260 (13)	10,940 (35)
Length of formal residency in Sweden				
Mean number of years (SD)		-	11.3 (3.4)	10.2 (4.4)
0-4 years	4,975 (0)	-	1,742 (4)	3,233 (10)
5-10 years	28,887 (3)	-	15,527 (39)	13,360 (42)
> 10 years	37,947 (4)	-	22,923 (57)	15,024 (47)
Missing	183 (0)	-	113 (0)	70 (0)
Work-related factors at baseline				
No unemployment	806,138 (80.3)	752,466 (80.8)	29,452 (73.1)	24,220 (76.4)
Unemployment ≤ 180 days/year	182,263 (18.2)	165,847 (17.8)	9,670 (24.0)	6,746 (21.3)
Unemployment >180days/year	15,359 (1.5)	13,455 (1.4)	1,183 (2.9)	721 (2.3)
No sickness absence (SA)	968,476 (96.5)	898,851 (96.5)	38,822 (96.3)	30,803 (97.2)
SA ≤90 days/year	25,798 (2.6)	24,078 (2.6)	1,094 (2.7)	626 (2.0)
SA >90 days/year	9,486 (0.9)	8,839 (0.9)	389 (1.0)	258 (0.8)
No disability pension (DP)	989,698 (98.6)	918,659 (98.6)	39,789 (98.7)	31,250 (98.6)
DP	13,762 (1.4)	12,809 (1.4)	516 (1.3)	437 (1.4)
Disorders at baseline				
Mental disorders[c]				
Any mental disorder	48,564 (4.8)	45,004 (4.8)	1,803 (4.5)	1,757 (5.5)
Substance misuse disorders	15,848 (1.6)	14,752 (1.6)	552 (1.4)	544 (1.7)
Schizophrenia/non-affective psychotic disorders	1,853 (0.2)	1,590 (0.2)	128 (0.3)	135 (0.4)
Depressive disorders	11,232 (1.1)	10,588 (1.1)	321 (0.8)	323 (1.0)
Anxiety disorders	9,040 (0.9)	8,520 (0.9)	286 (0.7)	234 (0.7)
Stress-related mental disorders	5,598 (0.6)	4,938 (0.55)	347 (0.9)	313 (1.0)
Behavioral disorders	9,016 (0.9)	8,492 (0.9)	232 (0.6)	292 (0.9)
Somatic disorders[c]				
Any somatic disorder	516,246 (51.4)	477,497 (51.2)	21,547 (53.5)	17,202 (54.3)
Infectious and parasitic diseases	39,940 (4.0)	36,433 (3.9)	1,922 (4.8)	1,585 (5.0)
Neoplasm	29,669 (3.0)	28,179 (3.0)	847 (2.1)	643 (2.0)
Diseases of the circulatory system	10,028 (1.0)	9,282 (1.0)	417 (1.0)	329 (1.0)
Diseases of the respiratory system	66,763 (6.7)	62,347 (6.7)	2,462 (6.1)	1,954 (6.2)
Diseases of the digestive system	52,144 (5.2)	48,135 (5.2)	2,299 (5.7)	1,710 (5.4)
Musculoskeletal diseases	65,865 (6.6)	61,347 (6.6)	2,530 (6.3)	1,988 (6.3)

For peer review only - http://bmjopen.bmj.com/site/about/guidelines.xhtml

Diseases of the nervous system	17,913 (1.8)	16,581 (1.8)	740 (1.8)	592 (1.9)
Other somatic diseases	419,249 (41.8)	386,602 (41.5)	18,028 (44.7)	14,619 (46.1)

[a] Living at home [b] Somalia, Eritrea and Ethiopia [c] During 2000-2004

Both refugees and non-refugee migrants had about a 20% lower risk of all-cause mortality and mortality due to external causes, while the risk of suicide was around 50% lower among non-refugee migrants and nearly 40% lower among refugees compared to Swedish-born individuals (Table 2). No differences between refugees and non-refugee migrants emerged

Table 2. *Risk of all-cause and cause-specific mortality in Swedish-born individuals, refugees and non-refugee migrants, aged 16-25 years old residing in Sweden in 2000-2004. Hazard ratios (HRs) with 95% confidence intervals (CIs).*

	N (rate per 100,000 person-years)	Model 1[a]	Model 2[b]
		**All-cause mortality**	
Swedish-born individuals	5,834 (53.4)	1 (REF)	1 (REF)
Non-refugee migrants	186 (52.1)	0.98 (0.85-1.13)	0.78 (0.67-0.91)
Refugees	229 (49.1)	0.92 (0.81-1.05)	0.80 (0.70-0.91)
		**Suicide**	
Swedish-born individuals	1,977 (18.1)	1 (REF)	1 (REF)
Non-refugee migrants	39 (10.9)	0.61 (0.44-0.83)	0.51 (0.37-0.70)
Refugees	60 (12.9)	0.71 (0.55-0.92)	0.63 (0.49-0.82)
		**External causes**	
Swedish-born individuals	3,873 (35.5)	1 (REF)	1 (REF)
Non-refugee migrants	123 (34.5)	0.97 (0.81-1.17)	0.78 (0.65-0.94)
Refugees	151 (32.4)	0.91 (0.78-1.08)	0.78 (0.66-0.92)

[a] Crude
[b] Adjusted for age, sex, education, family situation, type of living area, unemployment, sickness absence, disability pension at baseline, and mental and somatic disorders in 2000-2004.

Compared to Swedish-born youth without any mental disorder, Swedish born individuals with mental disorders had higher point estimates of all-cause mortality (HR: 3.4) as well as for death by suicide (HR: 5.8) and external causes (HR: 5.0). These estimates were lower in both non-refugee migrants (HRs: 2.4, 2.6 and 3.0, respectively) and refugees (HRs: 2.9, 3.8 and 4.4, respectively; Table 3). The same pattern, but less pronounced, was seen among those with somatic disorders, i.e., Swedish-born with somatic disorders had higher point estimates for all-cause mortality (HR: 1.6), suicide (HR: 1.3) and death due to external causes (HR: 1.4) compared to Swedish-born without somatic disorders. These estimates were again lower in

For peer review only - http://bmjopen.bmj.com/site/about/guidelines.xhtml

For peer review only

migrants: non-refugee migrants (HRs: 1.3, 0.7 and 1.2, respectively) and refugees (HRs: 1.3, 0.8 and 1.1, respectively).

Table 3. *Associations between mental and somatic disorders respectively, and all-cause and cause-specific mortality in Swedish-born individuals and refugee and non-refugee migrants, aged 16-25 years old residing in Sweden in 2000-2004. Hazard ratios (HRs) with 95% confidence intervals (CIs).*

**Refugee status**	**Mental/Somatic disorder status**	**n (rate per 100,000 person-years)**	**Model 1[a]**	**Model 2[b]**
			**All-cause mortality**	
Swedish-born individuals	No mental disorder	4,605 (44.3)	1 (REF)	1 (REF)
	Mental disorder	1,229 (234.4)	5.29 (4.97-5.64)	3.38 (3.16-3.62)
Non-refugee migrants	No mental disorder	149 (44.3)	1.00 (0.85-1.18)	0.81 (0.68-0.95)
	Mental disorder	37 (183.5)	4.15 (3.00-5.74)	2.35 (1.70-3.25)
Refugees	No mental disorder	178 (40.0)	0.90 (0.78-1.05)	0.78 (0.67-0.90)
	Mental disorder	51 (245.4)	5.55 (4.21-7.31)	2.94 (2.23-3.88)
			**Suicide**	
Swedish-born individuals	No mental disorder	1,434 (13.8)	1 (REF)	1 (REF)
	Mental disorder	543 (103.5)	7.51 (6.80-8.29)	5.84 (5.24-6.51)
Non-refugee migrants	No mental disorder	29 (8.6)	0.63 (0.43-0.91)	0.54 (0.37-0.78)
	Mental disorder	10 (49.6)	3.60 (1.93-6.71)	2.57 (1.38-4.81)
Refugees	No mental disorder	44 (9.9)	0.72 (0.53-0.97)	0.63 (0.46-0.85)
	Mental disorder	16 (77.0)	5.60 (3.42-9.16)	3.76 (2.29-6.18)
			**External causes**	
Swedish-born individuals	No mental disorder	2,912 (28.0)	1 (REF)	1 (REF)
	Mental disorder	961 (183.3)	6.55 (6.08-7.04)	5.02 (4.64-5.43)
Non-refugee migrants	No mental disorder	98 (29.1)	1.04 (0.85-1.27)	0.85 (0.69-1.04)
	Mental disorder	25 (124.0)	4.43 (2.99-6.57)	3.01 (2.03-4.48)
Refugees	No mental disorder	111 (24.9)	0.89 (0.74-1.08)	0.76 (0.62-0.91)
	Mental disorder	40 (192.5)	6.88 (5.04-9.40)	4.37 (3.19-5.98)
			**All-cause mortality**	
Swedish-born individuals	No somatic disorder	2,003 (37.7)	1 (REF)	1 (REF)
	Somatic disorder	3,831 (68.4)	1.82 (1.72-1.92)	1.59 (1.51-1.68)
Non-refugee migrants	No somatic disorder	55 (34.4)	0.92 (0.70-1.20)	0.71 (0.54-0.93)
	Somatic disorder	131 (66.6)	1.77 (1.48-2.11)	1.30 (1.08-1.55)
Refugees	No somatic disorder	74 (34.3)	0.91 (0.72-1.15)	0.82 (0.65-1.03)
	Somatic disorder	155 (62.0)	1.65 (1.40-1.94)	1.25 (1.06-1.48)
			**Suicide**	
Swedish-born individuals	No somatic disorder	758 (14.3)	1 (REF)	1 (REF)
	Somatic disorder	1,219 (21.8)	1.53 (1.39-1.67)	1.28 (1.16-1.40)
Non-refugee migrants	No somatic disorder	13 (8.1)	0.57 (0.33-0.99)	0.47 (0.27-0.81)
	Somatic disorder	26 (13.2)	0.93 (0.63-1.37)	0.68 (0.46-1.01)
Refugees	No somatic disorder	21 (9.7)	0.68 (0.44-1.05)	0.63 (0.41-0.97)
	Somatic disorder	39 (15.6)	1.10 (0.79-1.51)	0.81 (0.59-1.12)
			**External causes**	
Swedish-born individuals	No somatic disorder	1,461 (27.5)	1 (REF)	1 (REF)
	Somatic disorder	2,412 (43.1)	1.57 (1.47-1.67)	1.35 (1.26-1.44)
Non-refugee migrants	No somatic disorder	36 (22.5)	0.82 (0.59-1.15)	0.64 (0.46-0.89)
	Somatic disorder	87 (44.2)	1.61 (1.30-2.00)	1.17 (0.94-1.45)
Refugees	No somatic disorder	51 (23.6)	0.86 (0.65-1.14)	0.77 (0.58-1.01)
	Somatic disorder	100 (40.0)	1.46 (1.19-1.78)	1.07 (0.87-1.31)

[a] Crude
[b] Adjusted for age, sex, education, family situation, type of living area, unemployment, sickness absence, and disability pension at baseline. For exposure groups related to mental disorders, model 2 also adjusted for somatic morbidity in 2000-2004, and for exposure groups related to somatic disorders, model 2 also adjusted for mental disorders in 2000-2004.

For peer review only - http://bmjopen.bmj.com/site/about/guidelines.xhtml

Similar patterns were also observed for specific mental and somatic disorders with two exceptions: refugees and Swedish-born individuals with substance misuse disorder and neoplasms had equal point estimates of mortality (Table 4). Mortality risk for specific disorders in refugees compared to Swedish-born without the specific disorders ranged from 0.9 for infectious and parasitic diseases to 5.3 for substance misuse disorder.

For peer review only

Table 4. *Associations between specific disorders (in where there are >10 cases in either refugees or non-refugee migrants) and all-cause mortality in Swedish-born individuals and refugee and non-refugee migrants, aged 16-25 years old residing in Sweden in 2000-2004. Hazard ratios (HRs) with 95% confidence intervals (CIs).*

**Refugee status**	**Mental disorder status**	**n (rate per 100,000 person-years)**	**Model 1[a]**	**Model 2[b]**
Swedish-born individuals	No substance misuse disorder	5,158 (48.0)	1 (REF)	1 (REF)
	Substance misuse disorder	676 (397.8)	8.30 (7.66-8.99)	5.14 (4.73-5.59)
Non-refugee migrants	No substance misuse disorder	167 (47.6)	1.00 (0.85-1.16)	0.79 (0.68-0.93)
	Substance misuse disorder	19 (307.0)	6.41 (4.09-10.06)	3.48 (2.21-5.46)
Refugees	No substance misuse disorder	197 (42.8)	0.89 (0.78-1.03)	0.77 (0.67-0.89)
	Substance misuse disorder	32 (512.8)	10.73 (7.58-15.18)	5.28 (3.73-7.49)
Swedish-born individuals	No infectious and parasitic disease	5,418 (51.6)	1 (REF)	1 (REF)
	Infectious and parasitic disease	416 (98.0)	1.90 (1.72-2.10)	1.71 (1.55-1.90)
Non-refugee migrants	No infectious and parasitic disease	168 (49.6)	0.96 (0.83-1.12)	0.77 (0.66-0.90)
	Infectious and parasitic disease	18 (100.1)	1.94 (1.22-3.09)	1.34 (0.84-2.12)
Refugees	No infectious and parasitic disease	215 (48.4)	0.94 (0.82-1.08)	0.82 (0.72-0.94)
	Infectious and parasitic disease	14 (63.0)	1.22 (0.72-2.06)	0.90 (0.53-1.53)
Swedish-born individuals	No neoplasm	5,532 (52.2)	1 (REF)	1 (REF)
	Neoplasm	302 (91.8)	1.76 (1.57-1.97)	1.98 (1.76-2.22)
Non-refugee migrants	No neoplasm	181 (51.8)	1.00 (0.86-1.15)	0.79 (0.68-0.92)
	Neoplasm	< 10	~~1.31 (0.55-3.16)~~	~~1.16 (0.48-2.78)~~
Refugees	No neoplasm	219 (48.0)	0.92 (0.80-1.05)	0.80 (0.70-0.92)
	Neoplasm	40 (102.3)	1.96 (1.06-3.65)	1.96 (1.05-3.64)
Swedish-born individuals	No disease of the respiratory system	5,235 (51.4)	1 (REF)	1 (REF)
	Disease of the respiratory system	599 (81.9)	1.60 (1.47-1.74)	1.49 (1.37-1.62)
Non-refugee migrants	No disease of the respiratory system	166 (49.6)	0.97 (0.83-1.13)	0.77 (0.66-0.90)
	Disease of the respiratory system	20 (89.4)	1.74 (1.12-2.70)	1.19 (0.77-1.85)
Refugees	No disease of the respiratory system	210 (48.0)	0.94 (0.81-1.07)	0.82 (0.71-0.94)
	Disease of the respiratory system	19 (66.8)	1.30 (0.83-2.04)	0.98 (0.62-1.53)
Swedish-born individuals	No disease of the digestive system	5,342 (51.6)	1 (REF)	1 (REF)
	Disease of the digestive system	492 (87.2)	1.69 (1.54-1.85)	1.49 (1.36-1.63)
Non-refugee migrants	No disease of the digestive system	173 (51.3)	1.00 (0.86-1.16)	0.79 (0.68-0.92)
	Disease of the digestive system	13 (66.2)	1.29 (0.75-2.22)	0.88 (0.51-1.52)
Refugees	No disease of the digestive system	207 (47.1)	0.91 (0.80-1.05)	0.80 (0.69-0.92)
	Disease of the digestive system	22 (82.7)	1.61 (1.06-2.44)	1.16 (0.76-1.76)
Swedish-born individuals	No musculoskeletal disease	5,296 (51.9)	1 (REF)	1 (REF)
	Musculoskeletal disease	538 (74.7)	1.44 (1.32-1.57)	1.31 (1.20-1.43)
Non-refugee migrants	No musculoskeletal disease	177 (53.0)	1.02 (0.88-1.19)	0.81 (0.70-0.94)
	Musculoskeletal disease	<10	~~0.76 (0.40-1.46)~~	~~0.55 (0.29-1.06)~~

For peer review only - http://bmjopen.bmj.com/site/about/guidelines.xhtml

Refugees	No musculoskeletal disease	208 (47.6)	0.92 (0.80-1.05)	0.80 (0.70-0.92)
	Musculoskeletal disease	21 (71.7)	1.38 (0.90-2.12)	1.00 (0.65-1.53)
Swedish-born individuals	No disease of the nervous system	5,387 (50.2)	1 (REF)	1 (REF)
	Disease of the nervous system	447 (231.0)	4.60 (4.18-5.07)	2.83 (2.56-3.13)
Non-refugee migrants	No disease of the nervous system	170 (48.6)	0.97 (0.83-1.13)	0.78 (0.67-0.91)
	Disease of the nervous system	16 (236.0)	4.71 (2.89-7.70)	2.45 (1.50-4.01)
Refugees	No disease of the nervous system	215 (47.0)	0.94 (0.82-1.07)	0.82 (0.71-0.94)
	Disease of the nervous system	14 (161.6)	3.22 (1.91-5.44)	1.77 (1.05-3.00)

[a] Crude
[b] Adjusted for age, sex, education, family situation, type of living area, unemployment, sickness absence, disability pension at baseline, and somatic disorders in 2000-2004.

Discussion

Main findings

To the best of our knowledge this is the first study analysing the all-cuase and cause specific mortality risk in young refuees and non-refuee migrants with specific mental and somatic disorders. Refugees and non-refugee migrants had, compared to Swedish-born individuals, a 20 percent lower risk of all-cause mortality and mortality due to external causes. The risk of suicide was even lower (50% lower among non-refugees and nearly 40% lower among refugees) in the multivariate analysis. Similar patterns were found for specific mental and somatic disorders, whith exception of substance misuse disorder and neoplasms, where refugees and Swedish-born individuals had similar risk estimates.

The lower mortality risk in young migrants compared to the host population is in line with previous studies including all age groups.[10 12 22] As reported previously we could not find any differences in estimates regarding mortality between refugees and non-refugee migrants.[10] This is at odds with the higher occurrence of traumatic experiences and consequently higher risk of mental disorders and worse health status in refugees published previously.[23 24] An explanation for the lack of differences in estimates between refugees and non-refugee migrants may therefore be related to the importance of post-migration factors for subsequent mortality risk.

For peer review only - http://bmjopen.bmj.com/site/about/guidelines.xhtml

Here, factors like common cultural features, discrimination and low labour market attachment may affect refugees and non-refugee migrants equally.

Several models have been put forward to explain the mortality advantage among migrants.[10 12] A positive health selection of migrants has yielded the highest explanatory value, but also the healthy out-migration theory, often named the salmon bias, and flaws in the registration of migrants may be other reasons for the mortality advantage among migrants.[12]. We could now also show a mortality advantage in young migrants despite an earlier report on a higher mortality risk in younger (paritcular children) versus older migrants.[12] This latter paper reported a U-shaped pattern of the relation between age and mortality risk in migrants. The explanation most in line with this observed pattern was an in-migration selection effect. Namely that children most often arriving with a caretaker might not be subject to health selection to the same extent as their adolescent and young adult counterparts. The U-shaped pattern might therefore be more explained by the composition of the young migrants than by pure age effects. In our study approximately half of the young migrants arrived to Sweden more than 10 years before baseline, i.e. during childhood and the other half more recently.

While estimates for all-cause mortality and mortality due to external causes were comparable, we found the suicide risk in the young migrants to be particularly low. This finding is in contrast to previous reports on an increased suicide risk in young asylum seeking minors,[25] but in line with studies on refugees with a residence permit where no excess risk in suicide has been reported.[26] An explanation for the low suicide risk in young migrants with residence permit compared to those still facing an asylum seeking process might be both the protective effect of receiving a residence permit and the culturally determined strong stigmatisation of suicide. Previous research from Denmark showed that long asylum-decision waiting periods were associated with an increased risk of psychiatric disorders.[27] Host countries are therefore

For peer review only - http://bmjopen.bmj.com/site/about/guidelines.xhtml

advised to be aware of the mental health development of migrants during the asylum seeking process.

This is the first study examining associations between mental and somatic disorders and subsequent all-cause and cause-specific mortality in young refugees and non-refugee migrants. Studies investigating these associations in the general population – without a focus on migrants – have shown that individuals with mental disorders have a considerable higher risk for death due to external causes and suicide.[15 28] This is also true for somatic disorders but to a lesser extent.[28] In the present study, young Swedish-born individuals with a mental disorder had a six and five-fold elevated risk for suicide and mortality due to external causes compared with their peers without a mental disorder, respectively. Also, the risk for preterm mortality was increased with 60% in Swedish born with specialised health care due to any somatic disorder compared to their counterparts without such health care. We can now further show that in the presence of a mental disorder, the risk of suicide and mortality due to external causes was lower in non-refugee migrants than in Swedish-born individuals. Similar patterns were found for somatic disorders, albeit with smaller risk differences. These findings are thought-provoking and counterintuitive, as migrants with a mental or somatic disorder treated in specialised healthcare might be expected to suffer from disorders with a higher medical severity than their Swedish-born counterparts. This hypothesis arises from the common knowledge regarding discrepancies in access to and acceptance of specialised healthcare as well as treatment gaps of migrants compared with the population in high income host countries.[18 29]

Two exception to this pattern occurred, however. Estimates for all-cause mortality were equally high for young refugees and Swedish-born with substance use disorders and cancer. These differences in patterns might be related to the high medical severity of these two disorders and the fact that culturally influenced resilience factors such as favourable health

For peer review only - http://bmjopen.bmj.com/site/about/guidelines.xhtml

behaviour and strong social ties might be less relevant in protecting against preterm mortality related to these disorders.

Methodological considerations

This study has several strengths, including the longitudinal population-based design and use of national registers with high completeness and validity. The large cohort size allowed for detailed analyses of different types of mental and somatic disorders, the ability to adjust for important confounders and a rare outcome. Nevertheless, there are limitations. Information on exposure relied solely on register data from specialised care, and thus we have likely captured the most severe cases. This may lead to misclassification of undiagnosed and/or untreated individuals as unexposed. Potential differences in healthcare-seeking behaviour in Swedish-born individuals and migrants may also affect our findings. Moreover, there was a lack of data on important covariates for exemple social conncectedness and health behaviour. Further, the rare outcome may lead to lower statistical power for some of the analyses.

Conclusions

The risk of all-cause mortality as well as specific mortality due to suicide and death due to external causes is lower among young refugees and non-refugee migrants compared to Swedish-born individuals. The lower rate of mortality of refugees can also be seen among those with specific mental and somatic disorders. With few exceptions, young migrants with specific mental and somatic disorders have a mortality advantage compared to their Swedish-born peers with the same disorders. Further research on protective factors is warranted.

Funding

The work was supported by the Swedish Research Council (grant number 2018-05783).

For peer review only - http://bmjopen.bmj.com/site/about/guidelines.xhtml

Conflict of interest

None declared.

Ethics approval

Ethical approval of this study was given by the Regional Ethical Board in Stockholm (2007/762-31). The Ethical board also waived the requirement of informed consent from the participants in this study.

Data sharing statement

These data cannot be made publicly available due to privacy regulations. According to the General Data Protection Regulation, the Swedish law SFS 2018:218, the Swedish Data Protection Act, the Swedish Ethical Review Act, and the Public Access to Information and Secrecy Act, these types of sensitive data can only be made available for specific purposes, including research, that meets the criteria for access to this type of sensitive and confidential data as determined by a legal review. Readers may contact Professor Kristina Alexanderson (kristina.alexanderson@ki.se) regarding the data.

Contributorship statement

EMR and EB designed the research. EB analysed the data. EMR and MH wrote the manuscript. All authors contributed to successive drafts of the manuscript and approved the final version.

For peer review only

References

1. United Nations. International Migration Report: Highlights (ST/ESA/SER.A/404). New York: United Nations, Department of Economic and Social Affairs, Population Division, 2017.
2. UNHCR. Global trends: Forced displacement in 2017. In: Refugees UNHCf, ed. Global Trends, 2018:76.
3. Swedish Migration Agency. Applications for asylum received 2000-2019 (https://www.migrationsverket.se/English/About-the-Migration-Agency/Statistics/Asylum.html). Stockholm, 2020.
4. Sarkadi A, Ådahl K, Stenvall E, et al. Teaching Recovery Techniques: evaluation of a group intervention for unaccompanied refugee minors with symptoms of PTSD in Sweden. *Eur Child Adolesc Psychiatry* 2017 doi: 10.1007/s00787-017-1093-9
5. Hodes M, Vasquez MM, Anagnostopoulos D, et al. Refugees in Europe: national overviews from key countries with a special focus on child and adolescent mental health. *Eur Child Adolesc Psychiatry* 2018;27(4):389-99. doi: 10.1007/s00787-017-1094-8
6. Björkenstam E, Helgesson M, Norredam M, et al. Differences in psychiatric care utilization between refugees, non-refugee migrants and Swedish-born youth. *Psychol Med* 2020:1-11. doi: 10.1017/s0033291720003190 [published Online First: 2020/09/12]
7. Patton GC, Coffey C, Sawyer SM, et al. Global patterns of mortality in young people: a systematic analysis of population health data. *Lancet* 2009;374(9693):881-92. doi: 10.1016/s0140-6736(09)60741-8 [published Online First: 2009/09/15]
8. Amin R, Helgesson M, Runeson B, et al. Suicide attempt and suicide in refugees in Sweden - a nationwide population-based cohort study. *Psychol Med* 2019:1-10. doi: 10.1017/s0033291719003167 [published Online First: 2019/12/21]
9. Björkenstam E, Helgesson M, Amin R, et al. Mental disorders, suicide attempt and suicide: differences in the association in refugees compared with Swedish-born individuals. *Br J Psychiatry* 2019:1-7. doi: 10.1192/bjp.2019.215 [published Online First: 2019/10/15]
10. Aldridge RW, Nellums LB, Bartlett S, et al. Global patterns of mortality in international migrants: a systematic review and meta-analysis. *Lancet* 2018;392(10164):2553-66. doi: 10.1016/s0140-6736(18)32781-8 [published Online First: 2018/12/12]
11. Helgesson M, Rahman S, Saboonchi F, et al. Disability pension and mortality in individuals with specific somatic and mental disorders: examining differences between refugees and Swedish-born individuals. *J Epidemiol Community Health* 2021 doi: 10.1136/jech-2019-213436 [published Online First: 2021/01/22]
12. Guillot M, Khlat M, Elo I, et al. Understanding age variations in the migrant mortality advantage: An international comparative perspective. *PLoS One* 2018;13(6):e0199669. doi: 10.1371/journal.pone.0199669 [published Online First: 2018/06/30]
13. Helgesson M, Johansson B, Nordquist T, et al. Healthy migrant effect in the Swedish context: a register-based, longitudinal cohort study. *BMJ open* 2019;9(3):e026972. doi: 10.1136/bmjopen-2018-026972 [published Online First: 2019/03/18]
14. Mittendorfer-Rutz E, Rasmussen F, Wasserman D. Familial clustering of suicidal behaviour and psychopathology in young suicide attempters. A register-based nested case control study. *Soc Psychiatry Psychiatr Epidemiol* 2008;43(1):28-36. doi: 10.1007/s00127-007-0266-0 [published Online First: 2007/10/16]
15. Hällgren J, Ösby U, Westman J, et al. Mortality trends in external causes of death in people with mental health disorders in Sweden, 1987-2010. *Scand J Public Health* 2019;47(2):121-26. doi: 10.1177/1403494818758912 [published Online First: 2018/03/02]
16. Björkenstam E, Helgesson M, Norredam M, et al. Common mental disorders among young refugees in Sweden: The role of education and duration of residency. *J Affect Disord* 2020;266:563-71. doi: 10.1016/j.jad.2020.02.015 [published Online First: 2020/02/15]

For peer review only - http://bmjopen.bmj.com/site/about/guidelines.xhtml

17. Hjern A. Migration and public health: Health in Sweden: The National Public Health Report 2012. Chapter 13. *Scand J Public Health* 2012;40(9 Suppl):255-67. doi: 10.1177/1403494812459610 [published Online First: 2012/12/19]
18. Kohlenberger J, Buber-Ennser I, Rengs B, et al. Barriers to health care access and service utilization of refugees in Austria: Evidence from a cross-sectional survey. *Health Policy* 2019;123(9):833-39. doi: 10.1016/j.healthpol.2019.01.014 [published Online First: 2019/03/18]
19. Hollander AC. Social inequalities in mental health and mortality among refugees and other immigrants to Sweden--epidemiological studies of register data. *Glob Health Action* 2013;6:21059. doi: 10.3402/gha.v6i0.21059 [published Online First: 2013/07/03]
20. Diaz E, Kumar BN, Gimeno-Feliu LA, et al. Multimorbidity among registered immigrants in Norway: the role of reason for migration and length of stay. *Trop Med Int Health* 2015;20(12):1805-14. doi: 10.1111/tmi.12615 [published Online First: 2015/10/02]
21. Thordardottir EB, Yin L, Hauksdottir A, et al. Mortality and major disease risk among migrants of the 1991-2001 Balkan wars to Sweden: A register-based cohort study. *PLoS Med* 2020;17(12):e1003392. doi: 10.1371/journal.pmed.1003392 [published Online First: 2020/12/02]
22. Patrick D, Gadeyne S. Adult Migrant Mortality Advantage in Belgium: Evidence Using Census and Register Data. *Population (English Edition, 2002-)* 2005;60(5/6):655-98. doi: 10.2307/4148188
23. Gilliver SC, Sundquist J, Li X, et al. Recent research on the mental health of immigrants to Sweden: a literature review. *Eur J Public Health* 2014;24 Suppl 1:72-9. doi: 10.1093/eurpub/cku101 [published Online First: 2014/08/12]
24. Bas-Sarmiento P, Saucedo-Moreno MJ, Fernandez-Gutierrez M, et al. Mental Health in Immigrants Versus Native Population: A Systematic Review of the Literature. *Arch Psychiatr Nurs* 2017;31(1):111-21. doi: 10.1016/j.apnu.2016.07.014 [published Online First: 2017/01/21]
25. Mittendorfer-Rutz E, Hagström A, Hollander AC. High Suicide Rates Among Unaccompanied Minors/Youth Seeking Asylum in Sweden. *Crisis* 2020;41(4):314-17. doi: 10.1027/0227-5910/a000636 [published Online First: 2019/12/21]
26. Amin R, Rahman S, Dorner TE, et al. Country of birth, time period of resettlement and subsequent treated common mental disorders in young refugees in Sweden. *Eur J Public Health* 2020 doi: 10.1093/eurpub/ckaa140 [published Online First: 2020/08/26]
27. Hvidtfeldt C, Petersen JH, Norredam M. Prolonged periods of waiting for an asylum decision and the risk of psychiatric diagnoses: a 22-year longitudinal cohort study from Denmark. *Int J Epidemiol* 2020;49(2):400-09. doi: 10.1093/ije/dyz091 [published Online First: 2019/05/21]
28. Hawton K, van Heeringen K. Suicide. *Lancet* 2009;373(9672):1372-81. doi: 10.1016/s0140-6736(09)60372-x [published Online First: 2009/04/21]
29. Graetz V, Rechel B, Groot W, et al. Utilization of health care services by migrants in Europe-a systematic literature review. *Br Med Bull* 2017;121(1):5-18. doi: 10.1093/bmb/ldw057 [published Online First: 2017/01/22]

For peer review only

STROBE Statement—checklist of items that should be included in reports of observational studies

	Item No	Recommendation	Page No
**Title and abstract**	1	(*a*) Indicate the study's design with a commonly used term in the title or the abstract	1-2
		(*b*) Provide in the abstract an informative and balanced summary of what was done and what was found	2
**Introduction**			
Background/rationale	2	Explain the scientific background and rationale for the investigation being reported	5
Objectives	3	State specific objectives, including any prespecified hypotheses	6-7
**Methods**			
Study design	4	Present key elements of study design early in the paper	7
Setting	5	Describe the setting, locations, and relevant dates, including periods of recruitment, exposure, follow-up, and data collection	7
Participants	6	(*a*) *Cohort study*—Give the eligibility criteria, and the sources and methods of selection of participants. Describe methods of follow-up *Case-control study*—Give the eligibility criteria, and the sources and methods of case ascertainment and control selection. Give the rationale for the choice of cases and controls *Cross-sectional study*—Give the eligibility criteria, and the sources and methods of selection of participants	7
		(*b*) *Cohort study*—For matched studies, give matching criteria and number of exposed and unexposed *Case-control study*—For matched studies, give matching criteria and the number of controls per case	7
Variables	7	Clearly define all outcomes, exposures, predictors, potential confounders, and effect modifiers. Give diagnostic criteria, if applicable	8-9
Data sources/ measurement	8*	For each variable of interest, give sources of data and details of methods of assessment (measurement). Describe comparability of assessment methods if there is more than one group	*8*
Bias	9	Describe any efforts to address potential sources of bias	
Study size	10	Explain how the study size was arrived at	7
Quantitative variables	11	Explain how quantitative variables were handled in the analyses. If applicable, describe which groupings were chosen and why	9-10
Statistical methods	12	(*a*) Describe all statistical methods, including those used to control for confounding	9-10
		(*b*) Describe any methods used to examine subgroups and interactions	9-10
		(*c*) Explain how missing data were addressed	9
		(*d*) *Cohort study*—If applicable, explain how loss to follow-up was addressed *Case-control study*—If applicable, explain how matching of cases and controls was addressed *Cross-sectional study*—If applicable, describe analytical methods taking account of sampling strategy	
		(*e*) Describe any sensitivity analyses	9-10

Continued on next page

For peer review only - http://bmjopen.bmj.com/site/about/guidelines.xhtml

**Results**			
Participants	13*	(a) Report numbers of individuals at each stage of study—eg numbers potentially eligible, examined for eligibility, confirmed eligible, included in the study, completing follow-up, and analysed	10-15
		(b) Give reasons for non-participation at each stage	
		(c) Consider use of a flow diagram	
Descriptive data	14*	(a) Give characteristics of study participants (eg demographic, clinical, social) and information on exposures and potential confounders	10-15
		(b) Indicate number of participants with missing data for each variable of interest	10-15
		(c) *Cohort study*—Summarise follow-up time (eg, average and total amount)	11-15
Outcome data	15*	*Cohort study*—Report numbers of outcome events or summary measures over time	*10-15*
		Case-control study—Report numbers in each exposure category, or summary measures of exposure	*10-15*
		Cross-sectional study—Report numbers of outcome events or summary measures	
Main results	16	(*a*) Give unadjusted estimates and, if applicable, confounder-adjusted estimates and their precision (eg, 95% confidence interval). Make clear which confounders were adjusted for and why they were included	10-15
		(*b*) Report category boundaries when continuous variables were categorized	10-15
		(*c*) If relevant, consider translating estimates of relative risk into absolute risk for a meaningful time period	10-15
Other analyses	17	Report other analyses done—eg analyses of subgroups and interactions, and sensitivity analyses	
**Discussion**			
Key results	18	Summarise key results with reference to study objectives	15-18
Limitations	19	Discuss limitations of the study, taking into account sources of potential bias or imprecision. Discuss both direction and magnitude of any potential bias	18
Interpretation	20	Give a cautious overall interpretation of results considering objectives, limitations, multiplicity of analyses, results from similar studies, and other relevant evidence	18
Generalisability	21	Discuss the generalisability (external validity) of the study results	18
**Other information**			
Funding	22	Give the source of funding and the role of the funders for the present study and, if applicable, for the original study on which the present article is based	18

*Give information separately for cases and controls in case-control studies and, if applicable, for exposed and unexposed groups in cohort and cross-sectional studies.

Note: An Explanation and Elaboration article discusses each checklist item and gives methodological background and published examples of transparent reporting. The STROBE checklist is best used in conjunction with this article (freely available on the Web sites of PLoS Medicine at http://www.plosmedicine.org/, Annals of Internal Medicine at http://www.annals.org/, and Epidemiology at http://www.epidem.com/). Information on the STROBE Initiative is available at www.strobe-statement.org.

For peer review only - http://bmjopen.bmj.com/site/about/guidelines.xhtml

BMJ Open

Mental and somatic disorders and the subsequent risk of all-cause and cause-specific mortality in refugees, non-refugee migrants and the Swedish-born youth. A population-based cohort study in Sweden

Journal:	*BMJ Open*
Manuscript ID	bmjopen-2021-054351.R1
Article Type:	Original research
Date Submitted by the Author:	17-Feb-2022

Complete List of Authors:	Helgesson, Magnus; Karolinska Institutet, Department of Clinical Neuroscience<br>Björkenstam, Emma; Karolinska Institutet, Department of Clinical Neuroscience; Uppsala University, Department of Neuroscience<br>Filatova, Svetlana; Karolinska Institutet, Department of Clinical Neuroscience<br>Rahman, Syed; Karolinska Institutet, Department of Clinical Neuroscience<br>Cullen, Alexis; Karolinska Institutet, Department of Clinical Neuroscience; King's College London, Department of Psychosis Studies<br>Dorner, Thomas; Karolinska Institutet, Department of Clinical Neuroscience; Karl-Landsteiner Institute for Health Promotion Research<br>Gémes, Katalin; Karolinska Institutet, Department of Clinical Neuroscience<br>Amin , Ridwanul; Karolinska Institute, Division of Insurance Medicine, Department of Clinical Neuroscience<br>Mittendorfer-Rutz, Ellenor; Karolinska Institutet, Department of Clinical Neuroscience

**Primary Subject Heading**:	Epidemiology
Secondary Subject Heading:	Public health
Keywords:	EPIDEMIOLOGY, Suicide & self-harm < PSYCHIATRY, PUBLIC HEALTH

SCHOLARONE™
Manuscripts

I, the Submitting Author has the right to grant and does grant on behalf of all authors of the Work (as defined in the below author licence), an exclusive licence and/or a non-exclusive licence for contributions from authors who are: i) UK Crown employees; ii) where BMJ has agreed a CC-BY licence shall apply, and/or iii) in accordance with the terms applicable for US Federal Government officers or employees acting as part of their official duties; on a worldwide, perpetual, irrevocable, royalty-free basis to BMJ Publishing Group Ltd ("BMJ") its licensees and where the relevant Journal is co-owned by BMJ to the co-owners of the Journal, to publish the Work in this journal and any other BMJ products and to exploit all rights, as set out in our licence.

The Submitting Author accepts and understands that any supply made under these terms is made by BMJ to the Submitting Author unless you are acting as an employee on behalf of your employer or a postgraduate student of an affiliated institution which is paying any applicable article publishing charge ("APC") for Open Access articles. Where the Submitting Author wishes to make the Work available on an Open Access basis (and intends to pay the relevant APC), the terms of reuse of such Open Access shall be governed by a Creative Commons licence – details of these licences and which Creative Commons licence will apply to this Work are set out in our licence referred to above.

Other than as permitted in any relevant BMJ Author's Self Archiving Policies, I confirm this Work has not been accepted for publication elsewhere, is not being considered for publication elsewhere and does not duplicate material already published. I confirm all authors consent to publication of this Work and authorise the granting of this licence.

For peer review only

Cover page

Mental and somatic disorders and the subsequent risk of all-cause and cause-specific mortality in refugees, non-refugee migrants and the Swedish-born youth. A population-based cohort study in Sweden

Magnus Helgesson[1], Emma Björkenstam[1,2], Svetlana Filatova[1], Syed Rahman[1], Alexis E. Cullen [1,3], Thomas E. Dorner [1,4], Katalin Gemes[1], Ridwanul Amin[1], Ellenor Mittendorfer-Rutz[1*]

1 Division of Insurance Medicine, Department of Clinical Neuroscience, Karolinska Institutet, SE-171 77 Stockholm, Sweden

2 Department of Neuroscience, Uppsala University, Uppsala, Sweden

3 Department of Psychosis Studies, Institute of Psychiatry, Psychology & Neuroscience, King's College London, UK

4 Karl-Landsteiner Institute for Health Promotion Research, 3454 Sitzenberg-Reidling, Austria.

Corresponding author:

Ellenor Mittendorfer-Rutz, Professor, PhD

Ellenor.mittendorfer-rutz@ki.se

Karolinska Institutet

Word count: 2931 (max 4000)

For peer review only

Abstract (max 300; now 311)

Introduction: The aims were to investigate the associations between specific mental and somatic disorders and subsequent all-cause and cause-specific mortality (suicide, external and other causes) in young refugees and non-refugee migrants compared to Swedish-born individuals of similar age. **Methods:** In this register-based cohort study, all 1,003,760 individuals (40,305 refugees, 31,687 non-refugee migrants as the exposure groups, and the rest as the Swedish-born comparison group), 16-25 years old, residing in Sweden on 31-Dec-2004 were included. These individuals were followed regarding the outcome of all-cause and cause-specific mortality (suicide and external causes) between 2005-2016. The study population was also stratified according to any use of specialised healthcare for mental or somatic diagnoses before baseline (2000-2004). Cox regression models yielding crude and multivariate hazard ratios (HR and aHR, respectively) with 95% confidence intervals (CI) were used to investigate the aforementioned associations. **Results:** A lower proportion of both refugees (12%) and non-refugee migrants (10%) had college/university education compared to the Swedish-born individuals (17%). The proportion of unemployed (>180 days) among refugees (2.3%) and non-refugees (2.9%) was higher than the Swedish-born (1.4%). Refugees and non-refugee migrants had about a 20% lower risk of all-cause mortality and external causes of mortality compared to Swedish-born individuals. An even greater reduction in suicide risk (aHR 0.51, 95% CI 0.37-0.70, and 0.63, 95% CI 0.49-0.82 for non-refugees and refugees, respectively) was found. When restricted to those with a mental or somatic disorder, a lower risk of both general and specific mortality was also found among both refugees and non-refugee migrants compared to Swedish-born individuals. Refugees had, however, equal point estimates of all-cause mortality associated with substance misuse disorder and neoplasms as their Swedish-born peers with these disorders. **Conclusions:** With few exceptions, young migrants with specific mental and somatic disorders have a mortality

For peer review only - http://bmjopen.bmj.com/site/about/guidelines.xhtml

advantage compared to their Swedish-born peers with the same disorders. Further research on protective factors is warranted.

For peer review only

For peer review only - http://bmjopen.bmj.com/site/about/guidelines.xhtml

What are the new findings?

Refugees and non-refugee migrants had about a 20 percent lower risk of all-cause mortality and mortality due to external causes compared to Swedish-born individuals, and an even greater reduction in suicide risk.

When restricted to those with a mental or somatic disorder, a lower risk of both general and specific mortality was also found among both refugees and non-refugee migrants compared to Swedish-born individuals.

Refugees had, however, equal point estimates of all-cause mortality associated with substance misuse disorder and neoplasms as their Swedish-born peers with these disorders.

What do the new findings imply?

Further research on protective factors is warranted.

For peer review only

For peer review only - http://bmjopen.bmj.com/site/about/guidelines.xhtml

Introduction

During the past few years, there has been a substantial increase in global migration. In 2017, there were 258 million migrants worldwide, including 25.4 million refugees.[1 2] Sweden is one of the largest European recipients of refugees, during the last two decades, more than three quarters of a million refugees sought asylum in Sweden.[3] About one third of them were children, and a large proportion arrived in Sweden unaccompanied by parents or relatives. This development has the potential to be an eminent public health challenge, as many of the young refugees have experienced traumatic events, such as bereavement and violence, during war.[3-5] For these reasons, studies report a higher risk for mental disorders, particularly post-traumatic stress disorders in young refugees compared to the host population.[6-12]

Despite the large numbers of young refugees worldwide and their higher morbidity risk, there is a scarcity of studies focussing on the most prominent health outcomes, i.e. mortality, in this group. Mortality is a relatively rare event among young persons in general and death are mainly attributable to external causes including suicide, injuries, accidents, and poisoning.[13-15] Studies investigating mortality across all age groups have reported a lower mortality risk in refugees compared to the host population.[16 17] This migrant mortality advantage has puzzled researchers as it occurs against a background of higher morbidity levels in these populations.[18] Explanatory models include not only in- and out-migration health selection, but also cultural features such as better health behaviour but also data artefacts, primarily due to misclassification of age, nationality and ethnicity.[16 18 19] There seems to be an age variation in this migrant mortality advantage, however, where protection from excess mortality does not appear to apply to children as it does among their older counterparts.[18] Young refugees might be at a particular mortality risk, in part, as their main causes of death, suicide and external causes, are strongly related to mental disorders, which are known to have an early age of

For peer review only - http://bmjopen.bmj.com/site/about/guidelines.xhtml

onset and high prevalence rates in young refugees.[20-22] It is therefore crucial to investigate the mortality risk in young refugees.

Besides these higher prevalence rates of mental disorders, the occurrence of some somatic disorders (any non-psychiatric disorder), including for example, infectious diseases as well as diseases of the respiratory system and the nervous system, appear to be prevalent in refugees.[17 23] Variation in the importance of specific mental disorders between adult refugees and Swedish-born regarding the subsequent risk of suicide mortality has also been recently reported.[15] Therefore, specific mental and somatic disorders may play a differential role for the mortality risk among young refugees compared to young individuals in the host population. However, such knowledge is lacking to date. Any such differences might arise from unmet healthcare and treatment needs of young refugees with specific disorders.[24] Here, adequate care in young refugees might be hampered by language barriers, differences in the clinical manifestation and the course of the underlying disease, as well as the lack of competence in transcultural medicine among health care staff in the host country.

Studies report differences in the prevalence of both mental and somatic disorders as well as multimorbidity between subgroups of migrants. [25 26] Refugees have a higher risk of having experienced traumatic events, which might put them at a higher risk of developing different disorders and subsequently premature mortality compared to other migrants.[27] On the other hand, refugees and non-refugee migrants from the same countries of birth might share common culturally determined features, including the perception of health care, acceptance of treatment and health behaviour. To be able to create tailor-made interventions, there is therefore a need to study different subgroups of migrants separately.

This study aims to address the aforementioned gaps in the literature by investigating if the risk of all-cause and cause-specific mortality differs in young refugees and non-refugee migrants compared to individuals in the host population of the same age. A further aim was to

For peer review only - http://bmjopen.bmj.com/site/about/guidelines.xhtml

investigate if the potential differences in all-cause and cause-specific mortality vary by a prior diagnosis of specific mental and somatic disorders.

For peer review only

For peer review only - http://bmjopen.bmj.com/site/about/guidelines.xhtml

Methods

Study population

A cohort study was conducted where the study population was defined as all individuals aged 16–25 years, residing in Sweden on 31 December 2004, i.e., born between 1979 and 1988 (n = 1,081,412). Only those with complete information on their reason for settlement in Sweden were included (n = 1,048,762). For the non-refugee migrants, we excluded those who did not come from the same countries as the refugees (n = 9,008). A futher 35,994 individuals were excluded because they did not reside in Sweden during the period 2000-2004, i.e., the period during which mental and somatic disorders were measured. The final study population comprised of 1,003,760 young individuals 16-25 years, including 40,305 refugees and 31,687 non-refugee migrants with residence permit(temporary residents for <365 days, asylum seekers and undocumented migrants do not have a personal identity number in Sweden, and therefore, their information is not available in the administrative registers). Individuals in the study population were then followed from 2005-2016 regarding the outcome measures (please see the section on outcome) by linking several nationwide registers.

Patient and Public Involvement

As this study builds on administrative registers, patients were not involved in the conduct of this study.

Registers

We used the unique (de-identified) Swedish personal identity number to link information from several population-based registers. The researchers had full access to these databases. The Longitudinal Integration Database for Health Insurance and Labour Market Studies (LISA) contains data on sociodemographic and labour market factors. The Longitudinal Database for Integration Studies (STATIV) holds migration-related information, including reasons for settlement (e.g., refugee status). The National Patient Register (NPR) includes information on inpatient healthcare since 1987, and for specialised out-patient care since

For peer review only - http://bmjopen.bmj.com/site/about/guidelines.xhtml

2001. Diagnoses in NPR are coded according to the International Classification of Diseases, version 10 (ICD-10). The Cause of Death Register comprises information on causes and date of deaths of Swedish residents since 1952 (and including deaths registered abroad since 2012).

For peer review only

For peer review only - http://bmjopen.bmj.com/site/about/guidelines.xhtml

Measures

Mental disorders

Mental disorders were defined as having a main diagnosis from specialised healthcare (either inpatient or specialised outpatient healthcare), as recorded in the NPR between 2000 and 2004, of the following disorders: substance misuse disorders (ICD-10: F10–19), schizophrenia/non-affective psychotic disorders (F20–29), depressive disorders (ICD-10: F32–34), anxiety disorders (ICD-10: F40–42), stress-related disorders (F43) and behavioural disorders (ICD-10: F50–59, F90–99).

Somatic disorders

Main diagnoses in specialised healthcare (2000-2004) for somatic disorders were divided into dichotomised variables of following groups of diagnoses, recorded according to the International Classification of Disorders version 10 (ICD-10): 1) infectious and parasitic diseases (ICD-10: A00-B99), 2) neoplasm (ICD-10: C00-D48), 3) diseases of the circulatory system (ICD-10: I00-I99), 4) diseases of the respiratory system (ICD-10: J00-J99), 5) diseases of the digestive system (ICD-10: K00-K93), 6) musculoskeletal diseases (ICD-10: M00-M99), 7) diseases of the nervous system (ICD-10: G00-G99) and 8) other somatic disorders (ICD-10: E00--E90, H01-H99, L00-L99, N00-T99).

Outcome measures

The outcome measures were defined as both all-cause and cause-specific mortality. More specifically, we studied external causes (ICD-10: V00-Y99, including both intentional and unintentional injuries) as one outcome, and within this group, suicide (ICD-10: X60-84, Y10-34) was examined separately.

Covariates

The covariates included in the analyses were 1) socio-demographic factors: sex, age, educational level, type of living area and family situation (all variables were measured at

For peer review only

baseline, i.e., 31st December 2004); 2) work-related factors: unemployment (0, 1-180, >180 days); sickness absence (0, 1-90, >90 days) and disability pension, all measured during 2004. Missing values in any covariates were treated as a separate category in the adjusted analyses. Categorisations of covariates are shown in Table 1.

Statistical analyses

Statistical analyses were conducted using SAS, v.9.4. Crude and multivariate analyses were performed using Cox regression models of time to diagnosis-specific and all-cause mortality during the follow-up. The proportional hazard assumption was verified graphically using log-minus-log plots (plotting the log-log transformation of the survival function against survival time) over the different categories for each of the covariates and exposure variables. We assessed person-years at risk by totalling the years that the individuals were living in Sweden during the follow-up period. Calendar day was used as the underlying time scale and age at study entry was adjusted for in the multivariate-adjusted models. The entry date was defined as January 1st, 2005, and the exit date as the date of the first outcome, date of emigration, or the end of follow-up on December 31st, 2016. First, risk estimates for refugees and non-refugee migrants were compared to those for Swedish-born regarding subsequent all-cause mortality and cause-specific mortality. The association of mental and somatic disorders and subsequent all-cause mortality with refugee status was then assessed. Here, analyses were performed for any mental or somatic disorders and then we conducted separate analyses for each diagnostic group (only specific disorders where number of cases exceeded ten either in refugees or non-refugee migrants are shown in tables). We examined the associations between migrant status and each outcome in one crude and one adjusted regression model. Model 2 was adjusted for age, sex, education, family situation, type of living area, unemployment, sickness absence and disability pension, and finally morbidity (i.e., mental and somatic disorders as described above) at baseline. Analyses investigating the association with mental disorders and subsequent mortality were adjusted for somatic comorbidity and vice versa.

For peer review only - http://bmjopen.bmj.com/site/about/guidelines.xhtml

For peer review only

Results are presented as crude and adjusted hazard ratios (HRs and aHRs) with 95% confidence intervals (CIs).

For peer review only

For peer review only - http://bmjopen.bmj.com/site/about/guidelines.xhtml

Results

Among refugees, the percentage of men (53%) was found comparable to both Swedish-born (51%) and non-refugee migrants (49%, Table 1). There was a higher percentage of Swedish-born individuals (17%) with college/university education compared to both refugees (12%) and non-refugee migrants (10%). Among the refugees, the majority came from Former Yugoslavian countries (44%), whereas non-refugee migrants primarily emigrated from Asian countries. Most of the migrants had lived in Sweden for more than 10 years (47-57%). The percentage of of individuals who were unemployed >180 days was more than double among migrants (2.3-2.9%) compared to Swedish-born (1.4%), yet there were no differences between the groups regarding the prevalence of sickness absence and granting of disability pension. The prevalence of any specialised health care use due to mental disorder was highest among non-refugee migrants (5.5%) and lowest among refugees (4.5%). Likewise, specialised heatlh care use due to a somatic disorder occurred most frequently in non-refugee migrants (54.5%) followed by refugees (53.5%) and Swedish-born individuals (51.2%).

Table 1. *Cohort and health-related characteristics, by refugee status, in individuals aged 16-25 years old residing in Sweden in 2000-2004.*

	**All**	**Swedish-born individuals n (column %)**	**Refugees n (column %)**	**Non-refugee migrants n (column %)**
All, (n, row percent)	1,003,760 (100)	931,768 (93)	40,305 (4)	31,687 (3)
Sociodemographic factors at baseline				
Sex				
Women	487,254 (49)	452,142 (49)	19,041 (47)	16,071 (51)
Men	516,506 (51)	479,626 (51)	21,264 (53)	15,616 (49)
Age group				
16-20	525,724 (52)	487,676 (52)	21,378 (53)	16,670 (53)
21-25	478,036 (48)	444,092 (48)	18,927 (47)	15,017 (47)
Mean age (years, SD)	20.3 (2.9)	20.3 (2.9)	20.3 (2.8)	20.4 (2.9)
Education (years)				
Compulsory school (<9)	408,595 (41)	374,906 (40)	18,354 (46)	15,335 (48)
High school (10–12)	407,911 (41)	383,312 (41)	14,848 (37)	9,751 (31)
College or university (>12)	162,977 (16)	154,907 (17)	4,994 (12)	3,076 (10)
Missing	24,277 (2)	18,643 (2)	2,109 (5)	3,525 (11)
Family situation				
Married/living with partner without children[a]	8,915 (1)	6,342 (1)	1,518 (4)	1,055 (3)
Married/living with partner with children[a]	33,653 (3)	28,629 (3)	2,371 (6)	2,653 (8)
Single/divorced/separated/widowed without children[a]	483,651 (48)	453,573 (49)	17,067 (42)	13,011 (41)
Single/divorced/separated/widowed with children[a]	9,694 (1)	8,437 (1)	608 (2)	649 (2)
Children (≤20 years old)[2]	467,847 (47)	434,787 (47)	18,741 (46)	14,319 (45)
Type of living area				
Big city area	349,066 (35)	313,513 (34)	17,908 (44)	17,645 (56)
Intermediate (>90,000 inhabitants)	381,280 (38)	355,443 (38)	16,121 (40)	9,716 (31)

For peer review only - http://bmjopen.bmj.com/site/about/guidelines.xhtml

Small (rural municipalities)	273,414 (27)	262,812 (28)	6,276 (16)	4,326 (14))
Country/region of birth				
Horn of Africa[b]	4,675 (0)	-	1,917 (5)	2,758 (9)
Afghanistan	1,124 (0)	-	329 (1)	795 (3)
Iraq	8,464 (1)	-	4,120 (10)	4,344 (14)
Iran	7,366 (1)	-	4,773 (12)	2,593 (8)
Syria	1,942 (0)	-	1,423 (4)	519 (2)
Other countries from Asia (except Afghanistan, Iraq, Iran, and Syria)	12,645 (1)	-	4,631 (11)	8,014 (25)
Former Yugoslavian countries	19,576 (2)	-	17,852 (44)	1,724 (5)
Other countries (except Horn of Africa, Asian countries and former Yugoslavian countries)*	16,200 (2)	-	5,260 (13)	10,940 (35)
Length of formal residency in Sweden				
Mean number of years (SD)		-	11.3 (3.4)	10.2 (4.4)
0-4 years	4,975 (0)	-	1,742 (4)	3,233 (10)
5-10 years	28,887 (3)	-	15,527 (39)	13,360 (42)
> 10 years	37,947 (4)	-	22,923 (57)	15,024 (47)
Missing	183 (0)	-	113 (0)	70 (0)
Work-related factors at baseline				
No unemployment	806,138 (80.3)	752,466 (80.8)	29,452 (73.1)	24,220 (76.4)
Unemployment ≤ 180 days/year	182,263 (18.2)	165,847 (17.8)	9,670 (24.0)	6,746 (21.3)
Unemployment >180days/year	15,359 (1.5)	13,455 (1.4)	1,183 (2.9)	721 (2.3)
No sickness absence (SA)	968,476 (96.5)	898,851 (96.5)	38,822 (96.3)	30,803 (97.2)
SA ≤90 days/year	25,798 (2.6)	24,078 (2.6)	1,094 (2.7)	626 (2.0)
SA >90 days/year	9,486 (0.9)	8,839 (0.9)	389 (1.0)	258 (0.8)
No disability pension (DP)	989,698 (98.6)	918,659 (98.6)	39,789 (98.7)	31,250 (98.6)
DP	13,762 (1.4)	12,809 (1.4)	516 (1.3)	437 (1.4)
Disorders at baseline				
Mental disorders[c]				
Any mental disorder	48,564 (4.8)	45,004 (4.8)	1,803 (4.5)	1,757 (5.5)
Substance misuse disorders	15,848 (1.6)	14,752 (1.6)	552 (1.4)	544 (1.7)
Schizophrenia/non-affective psychotic disorders	1,853 (0.2)	1,590 (0.2)	128 (0.3)	135 (0.4)
Depressive disorders	11,232 (1.1)	10,588 (1.1)	321 (0.8)	323 (1.0)
Anxiety disorders	9,040 (0.9)	8,520 (0.9)	286 (0.7)	234 (0.7)
Stress-related mental disorders	5,598 (0.6)	4,938 (0.55)	347 (0.9)	313 (1.0)
Behavioral disorders	9,016 (0.9)	8,492 (0.9)	232 (0.6)	292 (0.9)
Somatic disorders[c]				
Any somatic disorder	516,246 (51.4)	477,497 (51.2)	21,547 (53.5)	17,202 (54.3)
Infectious and parasitic diseases	39,940 (4.0)	36,433 (3.9)	1,922 (4.8)	1,585 (5.0)
Neoplasm	29,669 (3.0)	28,179 (3.0)	847 (2.1)	643 (2.0)
Diseases of the circulatory system	10,028 (1.0)	9,282 (1.0)	417 (1.0)	329 (1.0)
Diseases of the respiratory system	66,763 (6.7)	62,347 (6.7)	2,462 (6.1)	1,954 (6.2)
Diseases of the digestive system	52,144 (5.2)	48,135 (5.2)	2,299 (5.7)	1,710 (5.4)
Musculoskeletal diseases	65,865 (6.6)	61,347 (6.6)	2,530 (6.3)	1,988 (6.3)
Diseases of the nervous system	17,913 (1.8)	16,581 (1.8)	740 (1.8)	592 (1.9)
Other somatic diseases	419,249 (41.8)	386,602 (41.5)	18,028 (44.7)	14,619 (46.1)

[a] Living at home [b] Somalia, Eritrea and Ethiopia [c] During 2000-2004; * other countries include primarily South American and remaining African countries

Both refugees and non-refugee migrants had about a 20% lower risk of all-cause mortality and mortality due to external causes, while the risk of suicide was around 50% lower among non-refugee migrants and nearly 40% lower among refugees compared to Swedish-born individuals (Table 2). No differences between refugees and non-refugee migrants emerged

Compared to Swedish-born youth without any mental disorder, Swedish born individuals with mental disorders had higher risk of all-cause mortality (aHR: 3.4, 95% CI: 3.2-3.6) as well as for death by suicide (aHR: 5.8, 95% CI: 5.2-6.5) and external causes (aHR: 5.0, 95% CI: 4.6-

For peer review only

For peer review only - http://bmjopen.bmj.com/site/about/guidelines.xhtml

5.4). These estimates were lower in both non-refugee migrants (aHRs: 2.4, 2.6 and 3.0, respectively) and refugees (aHRs: 2.9, 3.8 and 4.4, respectively; Table 3). The same pattern, but less pronounced, was seen among those with somatic disorders, i.e., Swedish-born with somatic disorders had higher risk for all-cause mortality (aHR: 1.6, 95% CI: 1.5-1.7), suicide (aHR: 1.3, 95% CI: 1.2-1.4) and death due to external causes (aHR: 1.4, 95% CI: 1.3-1.4) compared to Swedish-born without somatic disorders. These estimates were again lower in migrants: non-refugee migrants (aHRs: 1.3, 0.7 and 1.2, respectively) and refugees (HRs: 1.3, 0.8 and 1.1, respectively).

Table 2. *Risk of all-cause and cause-specific mortality in Swedish-born individuals, refugees and non-refugee migrants, aged 16-25 years old residing in Sweden in 2000-2004. Hazard ratios (HRs) with 95% confidence intervals (CIs).*

	N (rate per 100,000 person-years)	Model 1[a]	Model 2[b]
	**All-cause mortality**		
Swedish-born individuals	5,834 (53.4)	1 (REF)	1 (REF)
Non-refugee migrants	186 (52.1)	0.98 (0.85-1.13)	0.78 (0.67-0.91)
Refugees	229 (49.1)	0.92 (0.81-1.05)	0.80 (0.70-0.91)
	**Suicide**		
Swedish-born individuals	1,977 (18.1)	1 (REF)	1 (REF)
Non-refugee migrants	39 (10.9)	0.61 (0.44-0.83)	0.51 (0.37-0.70)
Refugees	60 (12.9)	0.71 (0.55-0.92)	0.63 (0.49-0.82)
	**External causes**		
Swedish-born individuals	3,873 (35.5)	1 (REF)	1 (REF)
Non-refugee migrants	123 (34.5)	0.97 (0.81-1.17)	0.78 (0.65-0.94)
Refugees	151 (32.4)	0.91 (0.78-1.08)	0.78 (0.66-0.92)

[a] Crude
[b] Adjusted for age, sex, education, family situation, type of living area, unemployment, sickness absence, disability pension at baseline, and mental and somatic disorders in 2000-2004.

Similar patterns were also observed for specific mental and somatic disorders with two exceptions: refugees and Swedish-born individuals with substance misuse disorder and neoplasms had equal point estimates of mortality (Table 4). Mortality risk for specific disorders in refugees compared to Swedish-born without the specific disorders ranged from 0.9 for infectious and parasitic diseases to 5.3 for substance misuse disorder.

For peer review only

Table 3. *Associations between mental and somatic disorders respectively, and all-cause and cause-specific mortality in Swedish-born individuals and refugee and non-refugee migrants, aged 16-25 years old residing in Sweden in 2000-2004. Hazard ratios (HRs) with 95% confidence intervals (CIs).*

**Mental/ disorder status**	**Refugee status**	**n (rate per 100,000 person-years)**	**Model 1a**	**Model 2b**
		**All-cause mortality**		
No mental disorder	Swedish-born	4,605 (44.3)	1 (REF)	1 (REF)
	Non-refugee migrants	149 (44.3)	1.00 (0.85-1.18)	**0.81 (0.68-0.95)**
	Refugees	178 (40.0)	0.90 (0.78-1.05)	**0.78 (0.67-0.90)**
Mental disorder	Swedish-born	1,229 (234.4)	**5.29 (4.97-5.64)**	**3.38 (3.16-3.62)**
	Non-refugee migrants	37 (183.5)	**4.15 (3.00-5.74)**	**2.35 (1.70-3.25)**
	Refugees	51 (245.4)	**5.55 (4.21-7.31)**	**2.94 (2.23-3.88)**
		**Suicide**		
No mental disorder	Swedish-born	1,434 (13.8)	1 (REF)	1 (REF)
	Non-refugee migrants	29 (8.6)	**0.63 (0.43-0.91)**	**0.54 (0.37-0.78)**
	Refugees	44 (9.9)	**0.72 (0.53-0.97)**	**0.63 (0.46-0.85)**
Mental disorder	Swedish-born	543 (103.5)	**7.51 (6.80-8.29)**	**5.84 (5.24-6.51)**
	Non-refugee migrants	10 (49.6)	**3.60 (1.93-6.71)**	**2.57 (1.38-4.81)**
	Refugees	16 (77.0)	**5.60 (3.42-9.16)**	**3.76 (2.29-6.18)**
		**External causes**		
No mental disorder	Swedish-born	2,912 (28.0)	1 (REF)	1 (REF)
	Non-refugee migrants	98 (29.1)	1.04 (0.85-1.27)	0.85 (0.69-1.04)
	Refugees	111 (24.9)	0.89 (0.74-1.08)	**0.76 (0.62-0.91)**
Mental disorder	Swedish-born	961 (183.3)	**6.55 (6.08-7.04)**	**5.02 (4.64-5.43)**
	Non-refugee migrants	25 (124.0)	**4.43 (2.99-6.57)**	**3.01 (2.03-4.48)**
	Refugees	40 (192.5)	**6.88 (5.04-9.40)**	**4.37 (3.19-5.98)**
**Somatic disorder status**				
		**All-cause mortality**		
No somatic disorder	Swedish-born	2,003 (37.7)	1 (REF)	1 (REF)
	Non-refugee migrants	55 (34.4)	0.92 (0.70-1.20)	**0.71 (0.54-0.93)**
	Refugees	74 (34.3)	0.91 (0.72-1.15)	0.82 (0.65-1.03)
Somatic disorder	Swedish-born	3,831 (68.4)	**1.82 (1.72-1.92)**	**1.59 (1.51-1.68)**
	Non-refugee migrants	131 (66.6)	**1.77 (1.48-2.11)**	**1.30 (1.08-1.55)**
	Refugees	155 (62.0)	**1.65 (1.40-1.94)**	**1.25 (1.06-1.48)**
		**Suicide**		
No somatic disorder	Swedish-born	758 (14.3)	1 (REF)	1 (REF)
	Non-refugee migrants	13 (8.1)	**0.57 (0.33-0.99)**	**0.47 (0.27-0.81)**
	Refugees	21 (9.7)	0.68 (0.44-1.05)	**0.63 (0.41-0.97)**
Somatic disorder	Swedish-born	1,219 (21.8)	**1.53 (1.39-1.67)**	**1.28 (1.16-1.40)**
	Non-refugee migrants	26 (13.2)	0.93 (0.63-1.37)	0.68 (0.46-1.01)
	Refugees	39 (15.6)	1.10 (0.79-1.51)	0.81 (0.59-1.12)
		**External causes**		
No somatic disorder	Swedish-born	1,461 (27.5)	1 (REF)	1 (REF)
	Non-refugee migrants	36 (22.5)	0.82 (0.59-1.15)	**0.64 (0.46-0.89)**
	Refugees	51 (23.6)	0.86 (0.65-1.14)	0.77 (0.58-1.01)
Somatic disorder	Swedish-born	2,412 (43.1)	**1.57 (1.47-1.67)**	**1.35 (1.26-1.44)**
	Non-refugee migrants	87 (44.2)	**1.61 (1.30-2.00)**	1.17 (0.94-1.45)
	Refugees	100 (40.0)	**1.46 (1.19-1.78)**	1.07 (0.87-1.31)

[a] Crude

[b] Adjusted for age, sex, education, family situation, type of living area, unemployment, sickness absence, and disability pension at baseline. For exposure groups related to mental disorders, model 2 was also adjusted for somatic morbidity in 2000-2004, and for exposure groups related to somatic disorders, model 2 was also adjusted for mental disorders in 2000-2004.

Values in bold are statistically significant associations (p-value <0.05)

For peer review only - http://bmjopen.bmj.com/site/about/guidelines.xhtml

Table 4. *Associations between specific disorders (in where there are >10 cases in either refugees or non-refugee migrants) and all-cause mortality in Swedish-born individuals and refugee and non-refugee migrants, aged 16-25 years old residing in Sweden in 2000-2004. Hazard ratios (HRs) with 95% confidence intervals (CIs).*

**Specific mental/somatic disorder status**	**Refugee status**	**n (rate per 100,000 person-years)**	**Model 1[a]**	**Model 2[b]**
No substance misuse disorder	Swedish-born	5,158 (48.0)	1 (REF)	1 (REF)
	Non-refugee migrants	167 (47.6)	1.00 (0.85-1.16)	**0.79 (0.68-0.93)**
	Refugees	197 (42.8)	0.89 (0.78-1.03)	**0.77 (0.67-0.89)**
Substance misuse disorder	Swedish-born	676 (397.8)	**8.30 (7.66-8.99)**	**5.14 (4.73-5.59)**
	Non-refugee migrants	19 (307.0)	**6.41 (4.09-10.06)**	**3.48 (2.21-5.46)**
	Refugees	32 (512.8)	**10.73 (7.58-15.18)**	**5.28 (3.73-7.49)**
No infectious and parasitic disease	Swedish-born	5,418 (51.6)	1 (REF)	1 (REF)
	Non-refugee migrants	168 (49.6)	0.96 (0.83-1.12)	**0.77 (0.66-0.90)**
	Refugees	215 (48.4)	0.94 (0.82-1.08)	**0.82 (0.72-0.94)**
Infectious and parasitic disease	Swedish-born	416 (98.0)	**1.90 (1.72-2.10)**	**1.71 (1.55-1.90)**
	Non-refugee migrants	18 (100.1)	**1.94 (1.22-3.09)**	1.34 (0.84-2.12)
	Refugees	14 (63.0)	1.22 (0.72-2.06)	0.90 (0.53-1.53)
No neoplasm	Swedish-born	5,532 (52.2)	1 (REF)	1 (REF)
	Non-refugee migrants	181 (51.8)	1.00 (0.86-1.15)	**0.79 (0.68-0.92)**
	Refugees	219 (48.0)	0.92 (0.80-1.05)	**0.80 (0.70-0.92)**
Neoplasm	Swedish-born	302 (91.8)	**1.76 (1.57-1.97)**	**1.98 (1.76-2.22)**
	Non-refugee migrants	< 10	1.31 (0.55-3.16)	1.16 (0.48-2.78)
	Refugees	40 (102.3)	**1.96 (1.06-3.65)**	**1.96 (1.05-3.64)**
No disease of the respiratory system	Swedish-born	5,235 (51.4)	1 (REF)	1 (REF)
	Non-refugee migrants	166 (49.6)	0.97 (0.83-1.13)	**0.77 (0.66-0.90)**
	Refugees	210 (48.0)	0.94 (0.81-1.07)	**0.82 (0.71-0.94)**
Disease of the respiratory system	Swedish-born	599 (81.9)	**1.60 (1.47-1.74)**	**1.49 (1.37-1.62)**
	Non-refugee migrants	20 (89.4)	**1.74 (1.12-2.70)**	1.19 (0.77-1.85)
	Refugees	19 (66.8)	1.30 (0.83-2.04)	0.98 (0.62-1.53)
No disease of the digestive system	Swedish-born	5,342 (51.6)	1 (REF)	1 (REF)
	Non-refugee migrants	173 (51.3)	1.00 (0.86-1.16)	**0.79 (0.68-0.92)**
	Refugees	207 (47.1)	0.91 (0.80-1.05)	**0.80 (0.69-0.92)**
Disease of the digestive system	Swedish-born	492 (87.2)	**1.69 (1.54-1.85)**	**1.49 (1.36-1.63)**
	Non-refugee migrants	13 (66.2)	1.29 (0.75-2.22)	0.88 (0.51-1.52)
	Refugees	22 (82.7)	**1.61 (1.06-2.44)**	1.16 (0.76-1.76)
No musculoskeletal disease	Swedish-born	5,296 (51.9)	1 (REF)	1 (REF)
	Non-refugee migrants	177 (53.0)	1.02 (0.88-1.19)	**0.81 (0.70-0.94)**
	Refugees	208 (47.6)	0.92 (0.80-1.05)	**0.80 (0.70-0.92)**
Musculoskeletal disease	Swedish-born	538 (74.7)	**1.44 (1.32-1.57)**	**1.31 (1.20-1.43)**
	Non-refugee migrants	<10	0.76 (0.40-1.46)	0.55 (0.29-1.06)
	Refugees	21 (71.7)	1.38 (0.90-2.12)	1.00 (0.65-1.53)
No disease of the nervous system	Swedish-born	5,387 (50.2)	1 (REF)	1 (REF)
	Non-refugee migrants	170 (48.6)	0.97 (0.83-1.13)	**0.78 (0.67-0.91)**
	Refugees	215 (47.0)	0.94 (0.82-1.07)	**0.82 (0.71-0.94)**
Disease of the nervous system	Swedish-born	447 (231.0)	**4.60 (4.18-5.07)**	**2.83 (2.56-3.13)**
	Non-refugee migrants	16 (236.0)	**4.71 (2.89-7.70)**	**2.45 (1.50-4.01)**
	Refugees	14 (161.6)	**3.22 (1.91-5.44)**	**1.77 (1.05-3.00)**

[a] Crude
[b] Adjusted for age, sex, education, family situation, type of living area, unemployment, sickness absence, disability pension at baseline, and somatic disorders in 2000-2004.
Values in bold are statistically significant associations (p-value <0.05)

For peer review only - http://bmjopen.bmj.com/site/about/guidelines.xhtml

Discussion

Main findings

Our study examined the all-cause and cause-specific mortality risk in young refuees and non-refugee migrants with specific mental and somatic disorders. Refugees and non-refugee migrants had, compared to Swedish-born individuals, a 20% lower risk of all-cause mortality and mortality due to external causes. The risk of suicide was even lower (50% lower among non-refugees and nearly 40% lower among refugees) in the multivariate analyses. Similar patterns were found for specific mental and somatic disorders, with the exception of substance misuse disorder and neoplasms, where refugees and Swedish-born individuals had similar risk estimates.

The lower mortality risk in young migrants compared to the host population is in line with previous studies including all age groups.[16 18 28] As reported previously we could not find any differences in estimates regarding mortality between refugees and non-refugee migrants.[16] This is at odds with the higher occurrence of traumatic experiences and consequently higher risk of mental disorders and worse health status in refugees published previously.[29 30] An explanation for the lack of differences in estimates between refugees and non-refugee migrants may therefore be related to the importance of post-migration factors for subsequent mortality risk. Here, factors like common cultural features, discrimination and low labour market attachment may affect refugees and non-refugee migrants equally.

Several models have been put forward to explain the mortality advantage among migrants.[16 18] A positive health selection of migrants ("healthy migrant effect") has yielded the highest explanatory value, but also the healthy out-migration theory, often named the salmon bias (potential return-migration to the country of birth when migrants are very sick), and flaws in the registration of migrants may be other reasons for the mortality advantage among migrants.[18]. We could now also show a mortality advantage in young migrants despite an

For peer review only - http://bmjopen.bmj.com/site/about/guidelines.xhtml

earlier report on a higher mortality risk in younger (paritcular children) versus older migrants.[18] This latter paper reported a U-shaped pattern of the relation between age and mortality risk in migrants. The explanation most in line with this observed pattern was an in-migration selection effect. Namely that children most often arriving with a caretaker might not be subject to health selection to the same extent as their adolescent and young adult counterparts. The U-shaped pattern might therefore be more explained by the composition of the young migrants than by pure age effects. In our study approximately half of the young migrants arrived to Sweden more than 10 years before baseline (i.e. during childhood) and the other half more recently. As our study population is young and we censored for emigration, salmon bias or faulty registration practices could be less likely explanations for the findings of mortality advantage among migrant youths over the Swedish-born youths in this study.

While estimates for all-cause mortality and mortality due to external causes were comparable, we found the suicide risk in the young migrants to be particularly low. This finding is in contrast to previous reports on an increased suicide risk in young asylum seeking minors,[31] but in line with studies on refugees with a residence permit where no excess risk in suicide has been reported.[32] An explanation for the low suicide risk in young migrants with residence permit compared to those still facing an asylum seeking process might be both the protective effect of receiving a residence permit and the culturally determined strong stigmatisation of suicide. Previous research from Denmark showed that long asylum-decision waiting periods were associated with an increased risk of psychiatric disorders.[33] Host countries are therefore advised to be aware of the mental health development of migrants during the asylum seeking process.

Our study examined the associations between mental and somatic disorders and subsequent all-cause and cause-specific mortality in young refugees and non-refugee migrants. Studies investigating these associations in the general population – without a focus on migrants –

For peer review only - http://bmjopen.bmj.com/site/about/guidelines.xhtml

have shown that individuals with mental disorders have a considerable higher risk for death due to external causes and suicide.[21 34] This is also true for somatic disorders but to a lesser extent.[34] In the present study, young Swedish-born individuals with a mental disorder had a six and five-fold elevated risk for suicide and mortality due to external causes compared with their peers without a mental disorder, respectively. Also, the risk for preterm mortality was increased with 60% in Swedish born with specialised health care due to any somatic disorder compared to their counterparts without such health care. We can now further show that in the presence of a mental disorder, the risk of suicide and mortality due to external causes was lower in non-refugee migrants than in Swedish-born individuals. Similar patterns were found for somatic disorders, albeit with smaller risk differences. These findings are thought-provoking and counterintuitive, as migrants with a mental or somatic disorder treated in specialised healthcare might be expected to suffer from disorders with a higher medical severity than their Swedish-born counterparts. This hypothesis arises from the common knowledge regarding discrepancies in access to and acceptance of specialised healthcare as well as treatment gaps of migrants compared with the population in high income host countries.[24 35]

Two exception to this pattern occurred, however. Estimates for all-cause mortality were equally high for young refugees and Swedish-born with substance use disorders and cancer. These differences in patterns might be related to the high medical severity of these two disorders and the fact that culturally influenced resilience factors such as favourable health behaviour and strong social ties might be less relevant in protecting against preterm mortality related to these disorders.

Methodological considerations

This study has several strengths, including the longitudinal population-based design and use of national registers with high completeness and validity. The large cohort size allowed for detailed analyses of different types of mental and somatic disorders, the ability to adjust for

For peer review only - http://bmjopen.bmj.com/site/about/guidelines.xhtml

important confounders and a rare outcome. Nevertheless, there are limitations. As information on primary healthcare was not available, we used data from specialised healthcare which captures only the more severe forms of morbidity. Due to different barriers to access healthcare that are specific to immigrants[36], we may have estimated differentially lower specialised healthcare use among refugees and non-refugee migrants than the Swedish-born. This may lead to misclassification of undiagnosed and/or untreated individuals as unexposed. Potential differences in healthcare-seeking behaviour in Swedish-born individuals and migrants may also affect our findings. Moreover, there was a lack of data on important covariates for example social connectedness and health behaviour. Furthermore, the rare outcome may lead to lower statistical power for some of the analyses. Additionally, generalisability of our results is limited to countries with similar healthcare and social welfare systems, and where the ethnic composition of the immigrant population is not significantly different than our study population. Moreover, these results can only be generalised to resettled refugees and non-refugee migrants dwelling in the community rather than those living in refugee camps or immigration detention centres.

Conclusions

The risk of all-cause mortality as well as specific mortality due to suicide and death due to external causes is lower among young refugees and non-refugee migrants compared to Swedish-born individuals. The lower rate of mortality of refugees can also be seen among those with specific mental and somatic disorders. With few exceptions, young migrants with specific mental and somatic disorders have a mortality advantage compared to their Swedish-born peers with the same disorders. Further research on protective factors is warranted.

For peer review only - http://bmjopen.bmj.com/site/about/guidelines.xhtml

Funding

The work was supported by the Swedish Research Council (grant number 2018-05783).

Conflict of interest

None declared.

Ethics approval

Ethical approval of this study was given by the Regional Ethical Board in Stockholm (2007/762-31). The Ethical board also waived the requirement of informed consent from the participants in this study.

Availability of data and material

These data cannot be made publicly available due to privacy regulations. According to the General Data Protection Regulation, the Swedish law SFS 2018:218, the Swedish Data Protection Act, the Swedish Ethical Review Act, and the Public Access to Information and Secrecy Act, these types of sensitive data can only be made available for specific purposes, including research, that meets the criteria for access to this type of sensitive and confidential data as determined by a legal review. Readers may contact Professor Kristina Alexanderson (kristina.alexanderson@ki.se) regarding the data.

It was not appropriate or possible to involve patients or the public in the design, or conduct, or reporting, or dissemination plans of our research.

Contributorship statement

The study was designed by MH, EB and EMR. EB conducted the data analyses. EMR and MH drafted the manuscript. SF, SR, AC, TD, KG and RA contributed with intellectual content and commented to results and successive drafts.

For peer review only

For peer review only - http://bmjopen.bmj.com/site/about/guidelines.xhtml

References

1. United Nations. International Migration Report: Highlights (ST/ESA/SER.A/404). New York: United Nations, Department of Economic and Social Affairs, Population Division, 2017.
2. UNHCR. Global trends: Forced displacement in 2017. In: Refugees UNHCf, ed. Global Trends, 2018:76.
3. Swedish Migration Agency. Applications for asylum received 2000-2019 (https://www.migrationsverket.se/English/About-the-Migration-Agency/Statistics/Asylum.html). Stockholm, 2020.
4. Sarkadi A, Ådahl K, Stenvall E, et al. Teaching Recovery Techniques: evaluation of a group intervention for unaccompanied refugee minors with symptoms of PTSD in Sweden. *European Child & Adolescent Psychiatry* 2017 doi: 10.1007/s00787-017-1093-9
5. Hodes M, Vasquez MM, Anagnostopoulos D, et al. Refugees in Europe: national overviews from key countries with a special focus on child and adolescent mental health. *European Child & Adolescent Psychiatry* 2018;27(4):389-99. doi: 10.1007/s00787-017-1094-8
6. Björkenstam E, Helgesson M, Norredam M, et al. Differences in psychiatric care utilization between refugees, non-refugee migrants and Swedish-born youth. *Psychol Med* 2020:1-11. doi: 10.1017/s0033291720003190 [published Online First: 2020/09/12]
7. Fazel M, Reed RV, Panter-Brick C, et al. Mental health of displaced and refugee children resettled in high-income countries: risk and protective factors. *The Lancet* 2012;379(9812):266-82. doi: 10.1016/s0140-6736(11)60051-2
8. Fazel M. Psychological and psychosocial interventions for refugee children resettled in high-income countries. *Epidemiology and psychiatric sciences* 2018;27(2):117-23. doi: 10.1017/S2045796017000695 [published Online First: 2017/11/10]
9. Norredam M, Nellums L, Nielsen RS, et al. Incidence of psychiatric disorders among accompanied and unaccompanied asylum-seeking children in Denmark: a nation-wide register-based cohort study. *European Child & Adolescent Psychiatry* 2018;27(4):439-46. doi: 10.1007/s00787-018-1122-3
10. Kien C, Sommer I, Faustmann A, et al. Prevalence of mental disorders in young refugees and asylum seekers in European Countries: a systematic review. *European Child & Adolescent Psychiatry* 2019;28(10):1295-310. doi: 10.1007/s00787-018-1215-z
11. Montgomery E. Trauma, exile and mental health in young refugees. *Acta psychiatrica Scandinavica* 2011;124(s440):1-46. doi: https://doi.org/10.1111/j.1600-0447.2011.01740.x
12. Manhica H, Almquist Y, Rostila M, et al. The use of psychiatric services by young adults who came to Sweden as teenage refugees: a national cohort study. *Epidemiology and psychiatric sciences* 2017;26(5):526-34. doi: 10.1017/s2045796016000445 [published Online First: 2016/06/30]
13. Patton GC, Coffey C, Sawyer SM, et al. Global patterns of mortality in young people: a systematic analysis of population health data. *Lancet* 2009;374(9693):881-92. doi: 10.1016/s0140-6736(09)60741-8 [published Online First: 2009/09/15]
14. Amin R, Helgesson M, Runeson B, et al. Suicide attempt and suicide in refugees in Sweden - a nationwide population-based cohort study. *Psychol Med* 2019:1-10. doi: 10.1017/s0033291719003167 [published Online First: 2019/12/21]
15. Björkenstam E, Helgesson M, Amin R, et al. Mental disorders, suicide attempt and suicide: differences in the association in refugees compared with Swedish-born individuals. *Br J Psychiatry* 2019:1-7. doi: 10.1192/bjp.2019.215 [published Online First: 2019/10/15]
16. Aldridge RW, Nellums LB, Bartlett S, et al. Global patterns of mortality in international migrants: a systematic review and meta-analysis. *Lancet* 2018;392(10164):2553-66. doi: 10.1016/s0140-6736(18)32781-8 [published Online First: 2018/12/12]
17. Helgesson M, Rahman S, Saboonchi F, et al. Disability pension and mortality in individuals with specific somatic and mental disorders: examining differences between refugees and

For peer review only - http://bmjopen.bmj.com/site/about/guidelines.xhtml

Swedish-born individuals. *J Epidemiol Community Health* 2021 doi: 10.1136/jech-2019-213436 [published Online First: 2021/01/22]
18. Guillot M, Khlat M, Elo I, et al. Understanding age variations in the migrant mortality advantage: An international comparative perspective. *PLoS One* 2018;13(6):e0199669. doi: 10.1371/journal.pone.0199669 [published Online First: 2018/06/30]
19. Helgesson M, Johansson B, Nordquist T, et al. Healthy migrant effect in the Swedish context: a register-based, longitudinal cohort study. *BMJ Open* 2019;9(3):e026972. doi: 10.1136/bmjopen-2018-026972 [published Online First: 2019/03/18]
20. Mittendorfer-Rutz E, Rasmussen F, Wasserman D. Familial clustering of suicidal behaviour and psychopathology in young suicide attempters. A register-based nested case control study. *Soc Psychiatry Psychiatr Epidemiol* 2008;43(1):28-36. doi: 10.1007/s00127-007-0266-0 [published Online First: 2007/10/16]
21. Hällgren J, Ösby U, Westman J, et al. Mortality trends in external causes of death in people with mental health disorders in Sweden, 1987-2010. *Scand J Public Health* 2019;47(2):121-26. doi: 10.1177/1403494818758912 [published Online First: 2018/03/02]
22. Björkenstam E, Helgesson M, Norredam M, et al. Common mental disorders among young refugees in Sweden: The role of education and duration of residency. *J Affect Disord* 2020;266:563-71. doi: 10.1016/j.jad.2020.02.015 [published Online First: 2020/02/15]
23. Hjern A. Migration and public health: Health in Sweden: The National Public Health Report 2012. Chapter 13. *Scand J Public Health* 2012;40(9 Suppl):255-67. doi: 10.1177/1403494812459610 [published Online First: 2012/12/19]
24. Kohlenberger J, Buber-Ennser I, Rengs B, et al. Barriers to health care access and service utilization of refugees in Austria: Evidence from a cross-sectional survey. *Health Policy* 2019;123(9):833-39. doi: 10.1016/j.healthpol.2019.01.014 [published Online First: 2019/03/18]
25. Hollander AC. Social inequalities in mental health and mortality among refugees and other immigrants to Sweden--epidemiological studies of register data. *Glob Health Action* 2013;6:21059. doi: 10.3402/gha.v6i0.21059 [published Online First: 2013/07/03]
26. Diaz E, Kumar BN, Gimeno-Feliu LA, et al. Multimorbidity among registered immigrants in Norway: the role of reason for migration and length of stay. *Trop Med Int Health* 2015;20(12):1805-14. doi: 10.1111/tmi.12615 [published Online First: 2015/10/02]
27. Thordardottir EB, Yin L, Hauksdottir A, et al. Mortality and major disease risk among migrants of the 1991-2001 Balkan wars to Sweden: A register-based cohort study. *PLoS medicine* 2020;17(12):e1003392. doi: 10.1371/journal.pmed.1003392 [published Online First: 2020/12/02]
28. Patrick D, Gadeyne S. Adult Migrant Mortality Advantage in Belgium: Evidence Using Census and Register Data. *Population (English Edition, 2002-)* 2005;60(5/6):655-98. doi: 10.2307/4148188
29. Gilliver SC, Sundquist J, Li X, et al. Recent research on the mental health of immigrants to Sweden: a literature review. *Eur J Public Health* 2014;24 Suppl 1:72-9. doi: 10.1093/eurpub/cku101 [published Online First: 2014/08/12]
30. Bas-Sarmiento P, Saucedo-Moreno MJ, Fernandez-Gutierrez M, et al. Mental Health in Immigrants Versus Native Population: A Systematic Review of the Literature. *Archives of psychiatric nursing* 2017;31(1):111-21. doi: 10.1016/j.apnu.2016.07.014 [published Online First: 2017/01/21]
31. Mittendorfer-Rutz E, Hagström A, Hollander AC. High Suicide Rates Among Unaccompanied Minors/Youth Seeking Asylum in Sweden. *Crisis* 2020;41(4):314-17. doi: 10.1027/0227-5910/a000636 [published Online First: 2019/12/21]
32. Amin R, Rahman S, Dorner TE, et al. Country of birth, time period of resettlement and subsequent treated common mental disorders in young refugees in Sweden. *Eur J Public Health* 2020 doi: 10.1093/eurpub/ckaa140 [published Online First: 2020/08/26]

For peer review only - http://bmjopen.bmj.com/site/about/guidelines.xhtml

33. Hvidtfeldt C, Petersen JH, Norredam M. Prolonged periods of waiting for an asylum decision and the risk of psychiatric diagnoses: a 22-year longitudinal cohort study from Denmark. *Int J Epidemiol* 2020;49(2):400-09. doi: 10.1093/ije/dyz091 [published Online First: 2019/05/21]
34. Hawton K, van Heeringen K. Suicide. *Lancet* 2009;373(9672):1372-81. doi: 10.1016/s0140-6736(09)60372-x [published Online First: 2009/04/21]
35. Graetz V, Rechel B, Groot W, et al. Utilization of health care services by migrants in Europe-a systematic literature review. *Br Med Bull* 2017;121(1):5-18. doi: 10.1093/bmb/ldw057 [published Online First: 2017/01/22]
36. Amin R, Rahman S, Tinghög P, et al. Healthcare use before and after suicide attempt in refugees and Swedish-born individuals. *Social psychiatry and psychiatric epidemiology* 2021;56:325-38. doi: 10.1007/s00127-020-01902-z

For peer review only

The RECORD statement – checklist of items, extended from the STROBE statement, that should be reported in observational studies using routinely collected health data.

	Item No.	STROBE items	Location in manuscript where items are reported	RECORD items	Location in manuscript where items are reported
**Title and abstract**					
	1	(a) Indicate the study's design with a commonly used term in the title or the abstract (b) Provide in the abstract an informative and balanced summary of what was done and what was found	(a) page 1<br>(b) page 2	RECORD 1.1: The type of data used should be specified in the title or abstract. When possible, the name of the databases used should be included.<br><br>RECORD 1.2: If applicable, the geographic region and timeframe within which the study took place should be reported in the title or abstract.<br><br>RECORD 1.3: If linkage between databases was conducted for the study, this should be clearly stated in the title or abstract.	Page 1<br>1.1: register data<br><br>1.2: Sweden<br><br>1.3: register-linkage<br><br>These information are mentioned in the title.
**Introduction**					
Background rationale	2	Explain the scientific background and rationale for the investigation being reported	Page 5-7		
Objectives	3	State specific objectives, including any prespecified hypotheses	Page 7		
**Methods**					
Study Design	4	Present key elements of study design early in the paper	Page 8		
Setting	5	Describe the setting, locations, and relevant dates, including periods of recruitment, exposure, follow-up, and data collection	Page 8-9		

For peer review only

Participants	6	*(a) Cohort study* - Give the eligibility criteria, and the sources and methods of selection of participants. Describe methods of follow-up<br>*Case-control study* - Give the eligibility criteria, and the sources and methods of case ascertainment and control selection. Give the rationale for the choice of cases and controls<br>*Cross-sectional study* - Give the eligibility criteria, and the sources and methods of selection of participants<br><br>*(b) Cohort study* - For matched studies, give matching criteria and number of exposed and unexposed<br>*Case-control study* - For matched studies, give matching criteria and the number of controls per case	Page 8	RECORD 6.1: The methods of study population selection (such as codes or algorithms used to identify subjects) should be listed in detail. If this is not possible, an explanation should be provided.<br><br>RECORD 6.2: Any validation studies of the codes or algorithms used to select the population should be referenced. If validation was conducted for this study and not published elsewhere, detailed methods and results should be provided.<br><br>RECORD 6.3: If the study involved linkage of databases, consider use of a flow diagram or other graphical display to demonstrate the data linkage process, including the number of individuals with linked data at each stage.	Page 6<br>6.1: All individuals who met the selection criteria were included from the nationwide registers. No algorithm was used.<br><br>6.2: Not applicable.<br><br>6.3: Using de-identified personal identity numbers, we linked the data for all individuals from the different registers. As there were no loss of individuals during the linking process, a flow-diagram was not deemed to be necessary.
Variables	7	Clearly define all outcomes, exposures, predictors, potential confounders, and effect modifiers. Give diagnostic criteria, if applicable.	Page 9-10	RECORD 7.1: A complete list of codes and algorithms used to classify exposures, outcomes, confounders, and effect modifiers should be provided. If these cannot be reported, an explanation should be provided.	Page
Data sources/ measurement	8	For each variable of interest, give sources of data and details	Page 8-10		

For peer review only

		of methods of assessment (measurement). Describe comparability of assessment methods if there is more than one group			
Bias	9	Describe any efforts to address potential sources of bias	-		
Study size	10	Explain how the study size was arrived at	Page 8		
Quantitative variables	11	Explain how quantitative variables were handled in the analyses. If applicable, describe which groupings were chosen, and why	Page 9-10		
Statistical methods	12	(a) Describe all statistical methods, including those used to control for confounding<br>(b) Describe any methods used to examine subgroups and interactions<br>(c) Explain how missing data were addressed<br>(d) *Cohort study* - If applicable, explain how loss to follow-up was addressed<br>*Case-control study* - If applicable, explain how matching of cases and controls was addressed<br>*Cross-sectional study* - If applicable, describe analytical methods taking account of sampling strategy<br>(e) Describe any sensitivity analyses	Page 10		
Data access and cleaning methods		..	Page 8	RECORD 12.1: Authors should describe the extent to which the investigators had access to the database	Page 8

For peer review only

				population used to create the study population.<br><br>RECORD 12.2: Authors should provide information on the data cleaning methods used in the study.	
Linkage		..	Page	RECORD 12.3: State whether the study included person-level, institutional-level, or other data linkage across two or more databases. The methods of linkage and methods of linkage quality evaluation should be provided.	Page 8
**Results**					
Participants	13	(a) Report the numbers of individuals at each stage of the study (*e.g.*, numbers potentially eligible, examined for eligibility, confirmed eligible, included in the study, completing follow-up, and analysed)<br>(b) Give reasons for non-participation at each stage.<br>(c) Consider use of a flow diagram	Page 8	RECORD 13.1: Describe in detail the selection of the persons included in the study (*i.e.,* study population selection) including filtering based on data quality, data availability and linkage. The selection of included persons can be described in the text and/or by means of the study flow diagram.	Page 8
Descriptive data	14	(a) Give characteristics of study participants (*e.g.*, demographic, clinical, social) and information on exposures and potential confounders<br>(b) Indicate the number of participants with missing data for each variable of interest<br>(c) *Cohort study* - summarise follow-up time (*e.g.*, average and total amount)	Page 11		

For peer review only

Outcome data	15	*Cohort study* - Report numbers of outcome events or summary measures over time<br>*Case-control study* - Report numbers in each exposure category, or summary measures of exposure<br>*Cross-sectional study* - Report numbers of outcome events or summary measures	Page 13		
Main results	16	(a) Give unadjusted estimates and, if applicable, confounder-adjusted estimates and their precision (e.g., 95% confidence interval). Make clear which confounders were adjusted for and why they were included<br>(b) Report category boundaries when continuous variables were categorized<br>(c) If relevant, consider translating estimates of relative risk into absolute risk for a meaningful time period	Page 12-15		
Other analyses	17	Report other analyses done—e.g., analyses of subgroups and interactions, and sensitivity analyses	Page ??		
**Discussion**					
Key results	18	Summarise key results with reference to study objectives	Page 16		
Limitations	19	Discuss limitations of the study, taking into account sources of potential bias or imprecision. Discuss both direction and magnitude of any potential bias	Page 18-19	RECORD 19.1: Discuss the implications of using data that were not created or collected to answer the specific research question(s). Include discussion of misclassification bias, unmeasured confounding, missing data, and changing eligibility over	Page 18-19

For peer review only

				time, as they pertain to the study being reported.	
Interpretation	20	Give a cautious overall interpretation of results considering objectives, limitations, multiplicity of analyses, results from similar studies, and other relevant evidence	Page 16-18		
Generalisability	21	Discuss the generalisability (external validity) of the study results	Page 19		
**Other Information**					
Funding	22	Give the source of funding and the role of the funders for the present study and, if applicable, for the original study on which the present article is based	Page 20		
Accessibility of protocol, raw data, and programming code		..		RECORD 22.1: Authors should provide information on how to access any supplemental information such as the study protocol, raw data, or programming code.	Page 20

*Reference: Benchimol EI, Smeeth L, Guttmann A, Harron K, Moher D, Petersen I, Sørensen HT, von Elm E, Langan SM, the RECORD Working Committee. The REporting of studies Conducted using Observational Routinely-collected health Data (RECORD) Statement. *PLoS Medicine* 2015; in press.

*Checklist is protected under Creative Commons Attribution (CC BY) license.

For peer review only - http://bmjopen.bmj.com/site/about/guidelines.xhtml

BMJ Open

Mental and somatic disorders and the subsequent risk of all-cause and cause-specific mortality in refugees, non-refugee migrants and the Swedish-born youth. A population-based cohort study in Sweden

Journal:	*BMJ Open*
Manuscript ID	bmjopen-2021-054351.R2
Article Type:	Original research
Date Submitted by the Author:	16-Apr-2022
Complete List of Authors:	Helgesson, Magnus; Karolinska Institutet, Department of Clinical Neuroscience<br>Björkenstam, Emma; Karolinska Institutet, Department of Clinical Neuroscience; Uppsala University, Department of Neuroscience<br>Filatova, Svetlana; Karolinska Institutet, Department of Clinical Neuroscience<br>Rahman, Syed; Karolinska Institutet, Department of Clinical Neuroscience<br>Cullen, Alexis; Karolinska Institutet, Department of Clinical Neuroscience; King's College London, Department of Psychosis Studies<br>Dorner, Thomas; Karolinska Institutet, Department of Clinical Neuroscience; Karl-Landsteiner Institute for Health Promotion Research<br>Gémes, Katalin; Karolinska Institutet, Department of Clinical Neuroscience<br>Amin , Ridwanul; Karolinska Institute, Division of Insurance Medicine, Department of Clinical Neuroscience<br>Mittendorfer-Rutz, Ellenor; Karolinska Institutet, Department of Clinical Neuroscience
**Primary Subject Heading**:	Epidemiology
Secondary Subject Heading:	Public health
Keywords:	EPIDEMIOLOGY, Suicide & self-harm < PSYCHIATRY, PUBLIC HEALTH

SCHOLARONE™
Manuscripts

I, the Submitting Author has the right to grant and does grant on behalf of all authors of the Work (as defined in the below author licence), an exclusive licence and/or a non-exclusive licence for contributions from authors who are: i) UK Crown employees; ii) where BMJ has agreed a CC-BY licence shall apply, and/or iii) in accordance with the terms applicable for US Federal Government officers or employees acting as part of their official duties; on a worldwide, perpetual, irrevocable, royalty-free basis to BMJ Publishing Group Ltd ("BMJ") its licensees and where the relevant Journal is co-owned by BMJ to the co-owners of the Journal, to publish the Work in this journal and any other BMJ products and to exploit all rights, as set out in our licence.

The Submitting Author accepts and understands that any supply made under these terms is made by BMJ to the Submitting Author unless you are acting as an employee on behalf of your employer or a postgraduate student of an affiliated institution which is paying any applicable article publishing charge ("APC") for Open Access articles. Where the Submitting Author wishes to make the Work available on an Open Access basis (and intends to pay the relevant APC), the terms of reuse of such Open Access shall be governed by a Creative Commons licence – details of these licences and which Creative Commons licence will apply to this Work are set out in our licence referred to above.

Other than as permitted in any relevant BMJ Author's Self Archiving Policies, I confirm this Work has not been accepted for publication elsewhere, is not being considered for publication elsewhere and does not duplicate material already published. I confirm all authors consent to publication of this Work and authorise the granting of this licence.

For peer review only

Cover page

Mental and somatic disorders and the subsequent risk of all-cause and cause-specific mortality in refugees, non-refugee migrants and the Swedish-born youth. A population-based cohort study in Sweden

Magnus Helgesson[1], Emma Björkenstam[1,2], Svetlana Filatova[1], Syed Rahman[1], Alexis E. Cullen [1,3], Thomas E. Dorner [1,4], Katalin Gemes[1], Ridwanul Amin[1], Ellenor Mittendorfer-Rutz[1*]

1 Division of Insurance Medicine, Department of Clinical Neuroscience, Karolinska Institutet, SE-171 77 Stockholm, Sweden

2 Department of Neuroscience, Uppsala University, Uppsala, Sweden

3 Department of Psychosis Studies, Institute of Psychiatry, Psychology & Neuroscience, King's College London, UK

4 Karl-Landsteiner Institute for Health Promotion Research, 3454 Sitzenberg-Reidling, Austria.

Corresponding author:

Ellenor Mittendorfer-Rutz, Professor, PhD

Ellenor.mittendorfer-rutz@ki.se

Karolinska Institutet

Word count: 2931 (max 4000)

For peer review only

Abstract (max 300; now 312)

Objectives: The aims were to investigate the associations between specific mental and somatic disorders and subsequent all-cause and cause-specific mortality (suicide, external and other causes) in young refugees and non-refugee migrants compared to Swedish-born individuals of similar age. **Methods:** In this register-based prospective cohort study, all 1,003,760 individuals (40,305 refugees, 31,687 non-refugee migrants as the exposure groups, and the rest as the Swedish-born comparison group), 16-25 years old, residing in Sweden on 31-Dec-2004 were included. These individuals were followed regarding the outcome of all-cause and cause-specific mortality (suicide and external causes) between 2005-2016. The study population was also stratified according to any use of specialised healthcare for mental or somatic diagnoses before baseline (2000-2004). Cox regression models yielding crude and multivariate hazard ratios (HR and aHR, respectively) with 95% confidence intervals (CI) were used to investigate the aforementioned associations. **Results:** A lower proportion of both refugees (12%) and non-refugee migrants (10%) had college/university education compared to the Swedish-born individuals (17%). The proportion of unemployed (>180 days) among refugees (2.3%) and non-refugees (2.9%) was higher than the Swedish-born (1.4%). Refugees and non-refugee migrants had about a 20% lower risk of all-cause mortality and external causes of mortality compared to Swedish-born individuals. An even greater reduction in suicide risk (aHR 0.51, 95% CI 0.37-0.70, and 0.63, 95% CI 0.49-0.82 for non-refugees and refugees, respectively) was found. When restricted to those with a mental or somatic disorder, a lower risk of both general and specific mortality was also found among both refugees and non-refugee migrants compared to Swedish-born individuals. Refugees had, however, equal point estimates of all-cause mortality associated with substance misuse disorder and neoplasms as their Swedish-born peers with these disorders. **Conclusions:** With few exceptions, young migrants with specific mental and somatic disorders have a mortality

advantage compared to their Swedish-born peers with the same disorders. Further research on protective factors is warranted.

Strengths and Limitations

- Longitudinal population-based design and use of national registers with high completeness and validity.
- Lack of data from primary healthcare may have led to differential underestimation of the mental and somatic disorders among migrant groups than Swedish-born host population.
- Generalisability is limited to countries with similar healthcare and social welfare systems, and populations with similar ethnic composition compared to Sweden.
- The results cannot be generalised to individuals living in refugee camps or immigration detention centres.

For peer review only

For peer review only - http://bmjopen.bmj.com/site/about/guidelines.xhtml

For peer review only

For peer review only - http://bmjopen.bmj.com/site/about/guidelines.xhtml

Introduction

During the past few years, there has been a substantial increase in global migration. In 2017, there were 258 million migrants worldwide, including 25.4 million refugees.[1 2] Sweden is one of the largest European recipients of refugees, during the last two decades, more than three quarters of a million refugees sought asylum in Sweden.[3] About one third of them were children, and a large proportion arrived in Sweden unaccompanied by parents or relatives. This development has the potential to be an eminent public health challenge, as many of the young refugees have experienced traumatic events, such as bereavement and violence, during war.[3-5] For these reasons, studies report a higher risk for mental disorders, particularly post-traumatic stress disorders in young refugees compared to the host population.[6-12]

Despite the large numbers of young refugees worldwide and their higher morbidity risk, there is a scarcity of studies focussing on the most prominent health outcomes, i.e. mortality, in this group. Mortality is a relatively rare event among young persons in general and death are mainly attributable to external causes including suicide, injuries, accidents, and poisoning.[13-15] Studies investigating mortality across all age groups have reported a lower mortality risk in refugees compared to the host population.[16 17] This migrant mortality advantage has puzzled researchers as it occurs against a background of higher morbidity levels in these populations.[18] Explanatory models include not only in- and out-migration health selection, but also cultural features such as better health behaviour but also data artefacts, primarily due to misclassification of age, nationality and ethnicity.[16 18 19] There seems to be an age variation in this migrant mortality advantage, however, where protection from excess mortality does not appear to apply to children as it does among their older counterparts.[18] Young refugees might be at a particular mortality risk, in part, as their main causes of death, suicide and external causes, are strongly related to mental disorders, which are known to have an early age of

For peer review only - http://bmjopen.bmj.com/site/about/guidelines.xhtml

onset and high prevalence rates in young refugees.[20-22] It is therefore crucial to investigate the mortality risk in young refugees.

Besides these higher prevalence rates of mental disorders, the occurrence of some somatic disorders (any non-psychiatric disorder), including for example, infectious diseases as well as diseases of the respiratory system and the nervous system, appear to be prevalent in refugees.[17 23] Variation in the importance of specific mental disorders between adult refugees and Swedish-born regarding the subsequent risk of suicide mortality has also been recently reported.[15] Therefore, specific mental and somatic disorders may play a differential role for the mortality risk among young refugees compared to young individuals in the host population. However, such knowledge is lacking to date. Any such differences might arise from unmet healthcare and treatment needs of young refugees with specific disorders.[24] Here, adequate care in young refugees might be hampered by language barriers, differences in the clinical manifestation and the course of the underlying disease, as well as the lack of competence in transcultural medicine among health care staff in the host country.

Studies report differences in the prevalence of both mental and somatic disorders as well as multimorbidity between subgroups of migrants. [25 26] Refugees have a higher risk of having experienced traumatic events, which might put them at a higher risk of developing different disorders and subsequently premature mortality compared to other migrants.[27] On the other hand, refugees and non-refugee migrants from the same countries of birth might share common culturally determined features, including the perception of health care, acceptance of treatment and health behaviour. To be able to create tailor-made interventions, there is therefore a need to study different subgroups of migrants separately.

This study aims to address the aforementioned gaps in the literature by investigating if the risk of all-cause and cause-specific mortality differs in young refugees and non-refugee migrants compared to individuals in the host population of the same age. A further aim was to

For peer review only - http://bmjopen.bmj.com/site/about/guidelines.xhtml

investigate if the potential differences in all-cause and cause-specific mortality vary by a prior diagnosis of specific mental and somatic disorders.

For peer review only

For peer review only - http://bmjopen.bmj.com/site/about/guidelines.xhtml

Methods

Study population

A cohort study was conducted where the study population was defined as all individuals aged 16–25 years, residing in Sweden on 31 December 2004, i.e., born between 1979 and 1988 (n = 1,081,412). Only those with complete information on their reason for settlement in Sweden were included (n = 1,048,762) because determining refugee vs non-refugee migrant status depends on the availability of this information. Individuals with incomplete information regarding this variable are primarily EU citizens who do not register their (and their families') right of residence with the Swedish Tax Agency. For the non-refugee migrants, we excluded those who did not come from the same countries as the refugees (n = 9,008) to investigate if traumatic experience as a refugee had affected mortality outcomes given that the post-migration conditions and the cultural background can be considered similar between refugees and non-refugee migrants from the same countries. A futher 35,994 individuals were excluded because they did not reside in Sweden during the period 2000-2004, i.e., the period during which mental and somatic disorders were measured. The final study population comprised of 1,003,760 young individuals 16-25 years, including 40,305 refugees and 31,687 non-refugee migrants with residence permit(temporary residents for <365 days, asylum seekers and undocumented migrants do not have a personal identity number in Sweden, and therefore, their information is not available in the administrative registers). Individuals in the study population were then followed from 2005-2016 regarding the outcome measures (please see the section on outcome) by linking several nationwide registers.

Patient and Public Involvement

As this study builds on administrative registers, patients were not involved in the conduct of this study.

For peer review only - http://bmjopen.bmj.com/site/about/guidelines.xhtml

Registers

We used the unique (de-identified) Swedish personal identity number to link information from several population-based registers. The researchers had full access to these databases. The Longitudinal Integration Database for Health Insurance and Labour Market Studies (LISA) contains data on sociodemographic and labour market factors. The Longitudinal Database for Integration Studies (STATIV) holds migration-related information, including reasons for settlement (e.g., refugee status). The National Patient Register (NPR) includes information on inpatient healthcare since 1987, and for specialised out-patient care since 2001. Diagnoses in NPR are coded according to the International Classification of Diseases, version 10 (ICD-10). The Cause of Death Register comprises information on causes and date of deaths of Swedish residents since 1952 (and including deaths registered abroad since 2012).

For peer review only - http://bmjopen.bmj.com/site/about/guidelines.xhtml

Measures

Mental disorders

Mental disorders were defined as having a main diagnosis from specialised healthcare (either inpatient or specialised outpatient healthcare), as recorded in the NPR between 2000 and 2004, of the following disorders: substance misuse disorders (ICD-10: F10–19), schizophrenia/non-affective psychotic disorders (F20–29), depressive disorders (ICD-10: F32–34), anxiety disorders (ICD-10: F40–42), stress-related disorders (F43) and behavioural disorders (ICD-10: F50–59, F90–99).

Somatic disorders

Main diagnoses in specialised healthcare (2000-2004) for somatic disorders were divided into dichotomised variables of following groups of diagnoses, recorded according to the International Classification of Disorders version 10 (ICD-10): 1) infectious and parasitic diseases (ICD-10: A00-B99), 2) neoplasm (ICD-10: C00-D48), 3) diseases of the circulatory system (ICD-10: I00-I99), 4) diseases of the respiratory system (ICD-10: J00-J99), 5) diseases of the digestive system (ICD-10: K00-K93), 6) musculoskeletal diseases (ICD-10: M00-M99), 7) diseases of the nervous system (ICD-10: G00-G99) and 8) other somatic disorders (ICD-10: E00--E90, H01-H99, L00-L99, N00-T99).

Outcome measures

The outcome measures were defined as both all-cause and cause-specific mortality. More specifically, we studied external causes (ICD-10: V00-Y99, including both intentional and unintentional injuries) as one outcome, and within this group, suicide (ICD-10: X60-84, Y10-34) was examined separately.

Covariates

The covariates included in the analyses were 1) socio-demographic factors: sex, age, educational level, type of living area and family situation (all variables were measured at

For peer review only - http://bmjopen.bmj.com/site/about/guidelines.xhtml

For peer review only

baseline, i.e., 31st December 2004); 2) work-related factors: unemployment (0, 1-180, >180 days); sickness absence (0, 1-90, >90 days) and disability pension, all measured during 2004. Missing values in any covariates were treated as a separate category in the adjusted analyses. Categorisations of covariates are shown in Table 1.

Statistical analyses

Statistical analyses were conducted using SAS, v.9.4. Crude and multivariate analyses were performed using Cox regression models of time to diagnosis-specific and all-cause mortality during the follow-up. The proportional hazard assumption was verified graphically using log-minus-log plots (plotting the log-log transformation of the survival function against survival time) over the different categories for each of the covariates and exposure variables. We assessed person-years at risk by totalling the years that the individuals were living in Sweden during the follow-up period. Calendar day was used as the underlying time scale and age at study entry was adjusted for in the multivariate-adjusted models. The entry date was defined as January 1st, 2005, and the exit date as the date of the first outcome, date of emigration, or the end of follow-up on December 31st, 2016. All individuals who emigrate are expected to notify the Swedish tax agency and this information is updated in all administrative registers. However, as it is possible that not everyone reports the emigration status, we additionally identified all individuals who did not have any information in the population register for two consecutive years and considered that they have emigrated during the former year (if they had not died before that) to compensate for the underreporting. First, risk estimates for refugees and non-refugee migrants were compared to those for Swedish-born regarding subsequent all-cause mortality and cause-specific mortality. The association of mental and somatic disorders and subsequent all-cause mortality with refugee status was then assessed. Here, analyses were performed for any mental or somatic disorders and then we conducted separate analyses for each diagnostic group (only specific disorders where number of cases exceeded ten either in refugees or non-refugee migrants are shown in tables). We examined the associations between

For peer review only - http://bmjopen.bmj.com/site/about/guidelines.xhtml

For peer review only

migrant status and each outcome in one crude and one adjusted regression model. Model 2 was adjusted for age, sex, education, family situation, type of living area, unemployment, sickness absence and disability pension, and finally morbidity (i.e., mental and somatic disorders as described above). Due to lack of data on time-dependent variables, we measured these covariates only at baseline. All covariates known to be associated with both the exposure and the outcome that do not fall in the causal pathway and for which data was available were included in the multivariate adjusted models. The directed acyclic graphs of unadjusted and adjusted models (please see supplementary material) did not reveal any biased pathway due to colliders. Moreover, multicollinearity among the covariates was tested using Cramér's V. Analyses investigating the association with mental dis orders and subsequent mortality were adjusted for somatic comorbidity and vice versa. The interaction between refugee status and mental/somatic disorders was investigated by stratified analyses. Results are presented as crude and adjusted hazard ratios (HRs and aHRs) with 95% confidence intervals (CIs).

For peer review only - http://bmjopen.bmj.com/site/about/guidelines.xhtml

Results

Among refugees, the percentage of men (53%) was found comparable to both Swedish-born (51%) and non-refugee migrants (49%, Table 1). There was a higher percentage of Swedish-born individuals (17%) with college/university education compared to both refugees (12%) and non-refugee migrants (10%). Among the refugees, the majority came from Former Yugoslavian countries (44%), whereas non-refugee migrants primarily emigrated from Asian countries. Most of the migrants had lived in Sweden for more than 10 years (47-57%). The percentage of of individuals who were unemployed >180 days was more than double among migrants (2.3-2.9%) compared to Swedish-born (1.4%), yet there were no differences between the groups regarding the prevalence of sickness absence and granting of disability pension. The prevalence of any specialised health care use due to mental disorder was highest among non-refugee migrants (5.5%) and lowest among refugees (4.5%). Likewise, specialised heatlh care use due to a somatic disorder occurred most frequently in non-refugee migrants (54.5%) followed by refugees (53.5%) and Swedish-born individuals (51.2%).

Table 1. *Cohort and health-related characteristics, by refugee status, in individuals aged 16-25 years old residing in Sweden in 2000-2004.*

	**All**	**Swedish-born individuals n (column %)**	**Refugees n (column %)**	**Non-refugee migrants n (column %)**
All, (n, row percent)	1,003,760 (100)	931,768 (93)	40,305 (4)	31,687 (3)
Sociodemographic factors at baseline				
Sex				
Women	487,254 (49)	452,142 (49)	19,041 (47)	16,071 (51)
Men	516,506 (51)	479,626 (51)	21,264 (53)	15,616 (49)
Age group				
16-20	525,724 (52)	487,676 (52)	21,378 (53)	16,670 (53)
21-25	478,036 (48)	444,092 (48)	18,927 (47)	15,017 (47)
Mean age (years, SD)	20.3 (2.9)	20.3 (2.9)	20.3 (2.8)	20.4 (2.9)
Education (years)				
Compulsory school (<9)	408,595 (41)	374,906 (40)	18,354 (46)	15,335 (48)
High school (10–12)	407,911 (41)	383,312 (41)	14,848 (37)	9,751 (31)
College or university (>12)	162,977 (16)	154,907 (17)	4,994 (12)	3,076 (10)
Missing	24,277 (2)	18,643 (2)	2,109 (5)	3,525 (11)
Family situation				
Married/living with partner without children[a]	8,915 (1)	6,342 (1)	1,518 (4)	1,055 (3)
Married/living with partner with children[a]	33,653 (3)	28,629 (3)	2,371 (6)	2,653 (8)
Single/divorced/separated/widowed without children[a]	483,651 (48)	453,573 (49)	17,067 (42)	13,011 (41)
Single/divorced/separated/widowed with children[a]	9,694 (1)	8,437 (1)	608 (2)	649 (2)
Children (≤20 years old)[2]	467,847 (47)	434,787 (47)	18,741 (46)	14,319 (45)
Type of living area				
Big city area	349,066 (35)	313,513 (34)	17,908 (44)	17,645 (56)
Intermediate (>90,000 inhabitants)	381,280 (38)	355,443 (38)	16,121 (40)	9,716 (31)

For peer review only - http://bmjopen.bmj.com/site/about/guidelines.xhtml

Small (rural municipalities)	273,414 (27)	262,812 (28)	6,276 (16)	4,326 (14))
Country/region of birth				
Horn of Africa[b]	4,675 (0)	-	1,917 (5)	2,758 (9)
Afghanistan	1,124 (0)	-	329 (1)	795 (3)
Iraq	8,464 (1)	-	4,120 (10)	4,344 (14)
Iran	7,366 (1)	-	4,773 (12)	2,593 (8)
Syria	1,942 (0)	-	1,423 (4)	519 (2)
Other countries from Asia (except Afghanistan, Iraq, Iran, and Syria)	12,645 (1)	-	4,631 (11)	8,014 (25)
Former Yugoslavian countries	19,576 (2)	-	17,852 (44)	1,724 (5)
Other countries (except Horn of Africa, Asian countries and former Yugoslavian countries)*	16,200 (2)	-	5,260 (13)	10,940 (35)
Length of formal residency in Sweden				
Mean number of years (SD)		-	11.3 (3.4)	10.2 (4.4)
0-4 years	4,975 (0)	-	1,742 (4)	3,233 (10)
5-10 years	28,887 (3)	-	15,527 (39)	13,360 (42)
> 10 years	37,947 (4)	-	22,923 (57)	15,024 (47)
Missing	183 (0)	-	113 (0)	70 (0)
Work-related factors at baseline				
No unemployment	806,138 (80.3)	752,466 (80.8)	29,452 (73.1)	24,220 (76.4)
Unemployment ≤ 180 days/year	182,263 (18.2)	165,847 (17.8)	9,670 (24.0)	6,746 (21.3)
Unemployment >180days/year	15,359 (1.5)	13,455 (1.4)	1,183 (2.9)	721 (2.3)
No sickness absence (SA)	968,476 (96.5)	898,851 (96.5)	38,822 (96.3)	30,803 (97.2)
SA ≤90 days/year	25,798 (2.6)	24,078 (2.6)	1,094 (2.7)	626 (2.0)
SA >90 days/year	9,486 (0.9)	8,839 (0.9)	389 (1.0)	258 (0.8)
No disability pension (DP)	989,698 (98.6)	918,659 (98.6)	39,789 (98.7)	31,250 (98.6)
DP	13,762 (1.4)	12,809 (1.4)	516 (1.3)	437 (1.4)
Disorders at baseline				
Mental disorders[c]				
Any mental disorder	48,564 (4.8)	45,004 (4.8)	1,803 (4.5)	1,757 (5.5)
Substance misuse disorders	15,848 (1.6)	14,752 (1.6)	552 (1.4)	544 (1.7)
Schizophrenia/non-affective psychotic disorders	1,853 (0.2)	1,590 (0.2)	128 (0.3)	135 (0.4)
Depressive disorders	11,232 (1.1)	10,588 (1.1)	321 (0.8)	323 (1.0)
Anxiety disorders	9,040 (0.9)	8,520 (0.9)	286 (0.7)	234 (0.7)
Stress-related mental disorders	5,598 (0.6)	4,938 (0.55)	347 (0.9)	313 (1.0)
Behavioral disorders	9,016 (0.9)	8,492 (0.9)	232 (0.6)	292 (0.9)
Somatic disorders[c]				
Any somatic disorder	516,246 (51.4)	477,497 (51.2)	21,547 (53.5)	17,202 (54.3)
Infectious and parasitic diseases	39,940 (4.0)	36,433 (3.9)	1,922 (4.8)	1,585 (5.0)
Neoplasm	29,669 (3.0)	28,179 (3.0)	847 (2.1)	643 (2.0)
Diseases of the circulatory system	10,028 (1.0)	9,282 (1.0)	417 (1.0)	329 (1.0)
Diseases of the respiratory system	66,763 (6.7)	62,347 (6.7)	2,462 (6.1)	1,954 (6.2)
Diseases of the digestive system	52,144 (5.2)	48,135 (5.2)	2,299 (5.7)	1,710 (5.4)
Musculoskeletal diseases	65,865 (6.6)	61,347 (6.6)	2,530 (6.3)	1,988 (6.3)
Diseases of the nervous system	17,913 (1.8)	16,581 (1.8)	740 (1.8)	592 (1.9)
Other somatic diseases	419,249 (41.8)	386,602 (41.5)	18,028 (44.7)	14,619 (46.1)

[a] Living at home [b] Somalia, Eritrea and Ethiopia [c] During 2000-2004; * other countries include primarily South American and remaining African countries

Both refugees and non-refugee migrants had about a 20% lower risk of all-cause mortality and mortality due to external causes, while the risk of suicide was around 50% lower among non-refugee migrants and nearly 40% lower among refugees compared to Swedish-born individuals (Table 2). No differences between refugees and non-refugee migrants emerged. The estimates in the multivariate models in these analyses were only to a minor extent altered by adjustment for the various covariates. The variable which contributed most to the changed estimates was age. Other variables only contributed marginally.

For peer review only

For peer review only - http://bmjopen.bmj.com/site/about/guidelines.xhtml

Compared to Swedish-born youth without any mental disorder, Swedish born individuals with mental disorders had higher risk of all-cause mortality (aHR: 3.4, 95% CI: 3.2-3.6) as well as for death by suicide (aHR: 5.8, 95% CI: 5.2-6.5) and external causes (aHR: 5.0, 95% CI: 4.6-5.4). These estimates were lower in both non-refugee migrants (aHRs: 2.4, 2.6 and 3.0, respectively) and refugees (aHRs: 2.9, 3.8 and 4.4, respectively; Table 3). The same pattern, but less pronounced, was seen among those with somatic disorders, i.e., Swedish-born with somatic disorders had higher risk for all-cause mortality (aHR: 1.6, 95% CI: 1.5-1.7), suicide (aHR: 1.3, 95% CI: 1.2-1.4) and death due to external causes (aHR: 1.4, 95% CI: 1.3-1.4) compared to Swedish-born without somatic disorders. These estimates were again lower in migrants: non-refugee migrants (aHRs: 1.3, 0.7 and 1.2, respectively) and refugees (HRs: 1.3, 0.8 and 1.1, respectively).

Table 2. *Risk of all-cause and cause-specific mortality in Swedish-born individuals, refugees and non-refugee migrants, aged 16-25 years old residing in Sweden in 2000-2004. Hazard ratios (HRs) with 95% confidence intervals (CIs).*

	N (rate per 100,000 person-years)	Model 1[a]	Model 2[b]
	**All-cause mortality**		
Swedish-born individuals	5,834 (53.4)	1 (REF)	1 (REF)
Non-refugee migrants	186 (52.1)	0.98 (0.85-1.13)	0.78 (0.67-0.91)
Refugees	229 (49.1)	0.92 (0.81-1.05)	0.80 (0.70-0.91)
	**Suicide**		
Swedish-born individuals	1,977 (18.1)	1 (REF)	1 (REF)
Non-refugee migrants	39 (10.9)	0.61 (0.44-0.83)	0.51 (0.37-0.70)
Refugees	60 (12.9)	0.71 (0.55-0.92)	0.63 (0.49-0.82)
	**External causes**		
Swedish-born individuals	3,873 (35.5)	1 (REF)	1 (REF)
Non-refugee migrants	123 (34.5)	0.97 (0.81-1.17)	0.78 (0.65-0.94)
Refugees	151 (32.4)	0.91 (0.78-1.08)	0.78 (0.66-0.92)

[a] Crude
[b] Adjusted for age, sex, education, family situation, type of living area, unemployment, sickness absence, disability pension at baseline, and mental and somatic disorders in 2000-2004.

Similar patterns were also observed for specific mental and somatic disorders with two exceptions: refugees and Swedish-born individuals with substance misuse disorder and neoplasms had equal point estimates of mortality (Table 4). Mortality risk for specific disorders in refugees compared to Swedish-born without the specific disorders ranged from 0.9 for infectious and parasitic diseases to 5.3 for substance misuse disorder.

For peer review only - http://bmjopen.bmj.com/site/about/guidelines.xhtml

Table 3. *Associations between mental and somatic disorders respectively, and all-cause and cause-specific mortality in Swedish-born individuals and refugee and non-refugee migrants, aged 16-25 years old residing in Sweden in 2000-2004. Hazard ratios (HRs) with 95% confidence intervals (CIs).*

**Mental/ disorder status**	**Refugee status**	**n (rate per 100,000 person-years)**	**Model 1[a]**	**Model 2[b]**
		**All-cause mortality**		
No mental disorder	Swedish-born	4,605 (44.3)	1 (REF)	1 (REF)
	Non-refugee migrants	149 (44.3)	1.00 (0.85-1.18)	**0.81 (0.68-0.95)**
	Refugees	178 (40.0)	0.90 (0.78-1.05)	**0.78 (0.67-0.90)**
Mental disorder	Swedish-born	1,229 (234.4)	**5.29 (4.97-5.64)**	**3.38 (3.16-3.62)**
	Non-refugee migrants	37 (183.5)	**4.15 (3.00-5.74)**	**2.35 (1.70-3.25)**
	Refugees	51 (245.4)	**5.55 (4.21-7.31)**	**2.94 (2.23-3.88)**
		**Suicide**		
No mental disorder	Swedish-born	1,434 (13.8)	1 (REF)	1 (REF)
	Non-refugee migrants	29 (8.6)	**0.63 (0.43-0.91)**	**0.54 (0.37-0.78)**
	Refugees	44 (9.9)	**0.72 (0.53-0.97)**	**0.63 (0.46-0.85)**
Mental disorder	Swedish-born	543 (103.5)	**7.51 (6.80-8.29)**	**5.84 (5.24-6.51)**
	Non-refugee migrants	10 (49.6)	**3.60 (1.93-6.71)**	**2.57 (1.38-4.81)**
	Refugees	16 (77.0)	**5.60 (3.42-9.16)**	**3.76 (2.29-6.18)**
		**External causes**		
No mental disorder	Swedish-born	2,912 (28.0)	1 (REF)	1 (REF)
	Non-refugee migrants	98 (29.1)	1.04 (0.85-1.27)	0.85 (0.69-1.04)
	Refugees	111 (24.9)	0.89 (0.74-1.08)	**0.76 (0.62-0.91)**
Mental disorder	Swedish-born	961 (183.3)	**6.55 (6.08-7.04)**	**5.02 (4.64-5.43)**
	Non-refugee migrants	25 (124.0)	**4.43 (2.99-6.57)**	**3.01 (2.03-4.48)**
	Refugees	40 (192.5)	**6.88 (5.04-9.40)**	**4.37 (3.19-5.98)**
**Somatic disorder status**				
		**All-cause mortality**		
No somatic disorder	Swedish-born	2,003 (37.7)	1 (REF)	1 (REF)
	Non-refugee migrants	55 (34.4)	0.92 (0.70-1.20)	**0.71 (0.54-0.93)**
	Refugees	74 (34.3)	0.91 (0.72-1.15)	0.82 (0.65-1.03)
Somatic disorder	Swedish-born	3,831 (68.4)	**1.82 (1.72-1.92)**	**1.59 (1.51-1.68)**
	Non-refugee migrants	131 (66.6)	**1.77 (1.48-2.11)**	**1.30 (1.08-1.55)**
	Refugees	155 (62.0)	**1.65 (1.40-1.94)**	**1.25 (1.06-1.48)**
		**Suicide**		
No somatic disorder	Swedish-born	758 (14.3)	1 (REF)	1 (REF)
	Non-refugee migrants	13 (8.1)	**0.57 (0.33-0.99)**	**0.47 (0.27-0.81)**
	Refugees	21 (9.7)	0.68 (0.44-1.05)	**0.63 (0.41-0.97)**
Somatic disorder	Swedish-born	1,219 (21.8)	**1.53 (1.39-1.67)**	**1.28 (1.16-1.40)**
	Non-refugee migrants	26 (13.2)	0.93 (0.63-1.37)	0.68 (0.46-1.01)
	Refugees	39 (15.6)	1.10 (0.79-1.51)	0.81 (0.59-1.12)
		**External causes**		
No somatic disorder	Swedish-born	1,461 (27.5)	1 (REF)	1 (REF)
	Non-refugee migrants	36 (22.5)	0.82 (0.59-1.15)	**0.64 (0.46-0.89)**
	Refugees	51 (23.6)	0.86 (0.65-1.14)	0.77 (0.58-1.01)
Somatic disorder	Swedish-born	2,412 (43.1)	**1.57 (1.47-1.67)**	**1.35 (1.26-1.44)**
	Non-refugee migrants	87 (44.2)	**1.61 (1.30-2.00)**	1.17 (0.94-1.45)
	Refugees	100 (40.0)	**1.46 (1.19-1.78)**	1.07 (0.87-1.31)

[a] Crude
[b] Adjusted for age, sex, education, family situation, type of living area, unemployment, sickness absence, and disability pension at baseline. For exposure groups related to mental disorders, model 2 was also adjusted for somatic morbidity in 2000-2004, and for exposure groups related to somatic disorders, model 2 was also adjusted for mental disorders in 2000-2004.
Values in bold are statistically significant associations (p-value <0.05)

For peer review only - http://bmjopen.bmj.com/site/about/guidelines.xhtml

Table 4. *Associations between specific disorders (in where there are >10 cases in either refugees or non-refugee migrants) and all-cause mortality in Swedish-born individuals and refugee and non-refugee migrants, aged 16-25 years old residing in Sweden in 2000-2004. Hazard ratios (HRs) with 95% confidence intervals (CIs).*

**Specific mental/somatic disorder status**	**Refugee status**	**n (rate per 100,000 person-years)**	**Model 1[a]**	**Model 2[b]**
No substance misuse disorder	Swedish-born	5,158 (48.0)	1 (REF)	1 (REF)
	Non-refugee migrants	167 (47.6)	1.00 (0.85-1.16)	**0.79 (0.68-0.93)**
	Refugees	197 (42.8)	0.89 (0.78-1.03)	**0.77 (0.67-0.89)**
Substance misuse disorder	Swedish-born	676 (397.8)	**8.30 (7.66-8.99)**	**5.14 (4.73-5.59)**
	Non-refugee migrants	19 (307.0)	**6.41 (4.09-10.06)**	**3.48 (2.21-5.46)**
	Refugees	32 (512.8)	**10.73 (7.58-15.18)**	**5.28 (3.73-7.49)**
No infectious and parasitic disease	Swedish-born	5,418 (51.6)	1 (REF)	1 (REF)
	Non-refugee migrants	168 (49.6)	0.96 (0.83-1.12)	**0.77 (0.66-0.90)**
	Refugees	215 (48.4)	0.94 (0.82-1.08)	**0.82 (0.72-0.94)**
Infectious and parasitic disease	Swedish-born	416 (98.0)	**1.90 (1.72-2.10)**	**1.71 (1.55-1.90)**
	Non-refugee migrants	18 (100.1)	**1.94 (1.22-3.09)**	1.34 (0.84-2.12)
	Refugees	14 (63.0)	1.22 (0.72-2.06)	0.90 (0.53-1.53)
No neoplasm	Swedish-born	5,532 (52.2)	1 (REF)	1 (REF)
	Non-refugee migrants	181 (51.8)	1.00 (0.86-1.15)	**0.79 (0.68-0.92)**
	Refugees	219 (48.0)	0.92 (0.80-1.05)	**0.80 (0.70-0.92)**
Neoplasm	Swedish-born	302 (91.8)	**1.76 (1.57-1.97)**	**1.98 (1.76-2.22)**
	Non-refugee migrants	< 10	1.31 (0.55-3.16)	1.16 (0.48-2.78)
	Refugees	40 (102.3)	**1.96 (1.06-3.65)**	**1.96 (1.05-3.64)**
No disease of the respiratory system	Swedish-born	5,235 (51.4)	1 (REF)	1 (REF)
	Non-refugee migrants	166 (49.6)	0.97 (0.83-1.13)	**0.77 (0.66-0.90)**
	Refugees	210 (48.0)	0.94 (0.81-1.07)	**0.82 (0.71-0.94)**
Disease of the respiratory system	Swedish-born	599 (81.9)	**1.60 (1.47-1.74)**	**1.49 (1.37-1.62)**
	Non-refugee migrants	20 (89.4)	**1.74 (1.12-2.70)**	1.19 (0.77-1.85)
	Refugees	19 (66.8)	1.30 (0.83-2.04)	0.98 (0.62-1.53)
No disease of the digestive system	Swedish-born	5,342 (51.6)	1 (REF)	1 (REF)
	Non-refugee migrants	173 (51.3)	1.00 (0.86-1.16)	**0.79 (0.68-0.92)**
	Refugees	207 (47.1)	0.91 (0.80-1.05)	**0.80 (0.69-0.92)**
Disease of the digestive system	Swedish-born	492 (87.2)	**1.69 (1.54-1.85)**	**1.49 (1.36-1.63)**
	Non-refugee migrants	13 (66.2)	1.29 (0.75-2.22)	0.88 (0.51-1.52)
	Refugees	22 (82.7)	**1.61 (1.06-2.44)**	1.16 (0.76-1.76)
No musculoskeletal disease	Swedish-born	5,296 (51.9)	1 (REF)	1 (REF)
	Non-refugee migrants	177 (53.0)	1.02 (0.88-1.19)	**0.81 (0.70-0.94)**
	Refugees	208 (47.6)	0.92 (0.80-1.05)	**0.80 (0.70-0.92)**
Musculoskeletal disease	Swedish-born	538 (74.7)	**1.44 (1.32-1.57)**	**1.31 (1.20-1.43)**
	Non-refugee migrants	<10	0.76 (0.40-1.46)	0.55 (0.29-1.06)
	Refugees	21 (71.7)	1.38 (0.90-2.12)	1.00 (0.65-1.53)
No disease of the nervous system	Swedish-born	5,387 (50.2)	1 (REF)	1 (REF)
	Non-refugee migrants	170 (48.6)	0.97 (0.83-1.13)	**0.78 (0.67-0.91)**
	Refugees	215 (47.0)	0.94 (0.82-1.07)	**0.82 (0.71-0.94)**
Disease of the nervous system	Swedish-born	447 (231.0)	**4.60 (4.18-5.07)**	**2.83 (2.56-3.13)**
	Non-refugee migrants	16 (236.0)	**4.71 (2.89-7.70)**	**2.45 (1.50-4.01)**
	Refugees	14 (161.6)	**3.22 (1.91-5.44)**	**1.77 (1.05-3.00)**

[a] Crude
[b] Adjusted for age, sex, education, family situation, type of living area, unemployment, sickness absence, disability pension at baseline, and somatic disorders in 2000-2004.
Values in bold are statistically significant associations (p-value <0.05)

For peer review only - http://bmjopen.bmj.com/site/about/guidelines.xhtml

Discussion

Main findings

Our study examined the all-cause and cause-specific mortality risk in young refuees and non-refugee migrants with specific mental and somatic disorders. Refugees and non-refugee migrants had, compared to Swedish-born individuals, a 20% lower risk of all-cause mortality and mortality due to external causes. The risk of suicide was even lower (50% lower among non-refugees and nearly 40% lower among refugees) in the multivariate analyses. Similar patterns were found for specific mental and somatic disorders, with the exception of substance misuse disorder and neoplasms, where refugees and Swedish-born individuals had similar risk estimates.

The lower mortality risk in young migrants compared to the host population is in line with previous studies including all age groups.[16 18 28] As reported previously we could not find any differences in estimates regarding mortality between refugees and non-refugee migrants.[16] This is at odds with the higher occurrence of traumatic experiences and consequently higher risk of mental disorders and worse health status in refugees published previously.[29 30] An explanation for the lack of differences in estimates between refugees and non-refugee migrants may therefore be related to the importance of post-migration factors for subsequent mortality risk. Here, factors like common cultural features, discrimination and low labour market attachment may affect refugees and non-refugee migrants equally.

Several models have been put forward to explain the mortality advantage among migrants.[16 18] A positive health selection of migrants ("healthy migrant effect") has yielded the highest explanatory value, but also the healthy out-migration theory, often named the salmon bias (potential return-migration to the country of birth when migrants are very sick), and flaws in the registration of migrants may be other reasons for the mortality advantage among migrants.[18]. We could now also show a mortality advantage in young migrants despite an

For peer review only - http://bmjopen.bmj.com/site/about/guidelines.xhtml

earlier report on a higher mortality risk in younger (paritcular children) versus older migrants.[18] This latter paper reported a U-shaped pattern of the relation between age and mortality risk in migrants. The explanation most in line with this observed pattern was an in-migration selection effect. Namely that children most often arriving with a caretaker might not be subject to health selection to the same extent as their adolescent and young adult counterparts. The U-shaped pattern might therefore be more explained by the composition of the young migrants than by pure age effects. In our study approximately half of the young migrants arrived to Sweden more than 10 years before baseline (i.e. during childhood) and the other half more recently. As our study population is young and we censored for emigration, salmon bias or faulty registration practices could be less likely explanations for the findings of mortality advantage among migrant youths over the Swedish-born youths in this study.

While estimates for all-cause mortality and mortality due to external causes were comparable, we found the suicide risk in the young migrants to be particularly low. This finding is in contrast to previous reports on an increased suicide risk in young asylum seeking minors,[31] but in line with studies on refugees with a residence permit where no excess risk in suicide has been reported.[32] An explanation for the low suicide risk in young migrants with residence permit compared to those still facing an asylum seeking process might be both the protective effect of receiving a residence permit and the culturally determined strong stigmatisation of suicide. Previous research from Denmark showed that long asylum-decision waiting periods were associated with an increased risk of psychiatric disorders.[33] Host countries are therefore advised to be aware of the mental health development of migrants during the asylum seeking process.

Our study examined the associations between mental and somatic disorders and subsequent all-cause and cause-specific mortality in young refugees and non-refugee migrants. Studies investigating these associations in the general population – without a focus on migrants –

For peer review only - http://bmjopen.bmj.com/site/about/guidelines.xhtml

have shown that individuals with mental disorders have a considerable higher risk for death due to external causes and suicide.[21 34] This is also true for somatic disorders but to a lesser extent.[34] In the present study, young Swedish-born individuals with a mental disorder had a six and five-fold elevated risk for suicide and mortality due to external causes compared with their peers without a mental disorder, respectively. Also, the risk for preterm mortality was increased with 60% in Swedish born with specialised health care due to any somatic disorder compared to their counterparts without such health care. We can now further show that in the presence of a mental disorder, the risk of suicide and mortality due to external causes was lower in non-refugee migrants than in Swedish-born individuals. Similar patterns were found for somatic disorders, albeit with smaller risk differences. These findings are thought-provoking and counterintuitive, as migrants with a mental or somatic disorder treated in specialised healthcare might be expected to suffer from disorders with a higher medical severity than their Swedish-born counterparts. This hypothesis arises from the common knowledge regarding discrepancies in access to and acceptance of specialised healthcare as well as treatment gaps of migrants compared with the population in high income host countries.[24 35]

Two exception to this pattern occurred, however. Estimates for all-cause mortality were equally high for young refugees and Swedish-born with substance use disorders and cancer. These differences in patterns might be related to the high medical severity of these two disorders and the fact that culturally influenced resilience factors such as favourable health behaviour and strong social ties might be less relevant in protecting against preterm mortality related to these disorders.

Methodological considerations

This study has several strengths, including the longitudinal population-based design and use of national registers with high completeness and validity. The large cohort size allowed for detailed analyses of different types of mental and somatic disorders, the ability to adjust for

For peer review only - http://bmjopen.bmj.com/site/about/guidelines.xhtml

important confounders and a rare outcome. Nevertheless, there are limitations. As information on primary healthcare was not available, we used data from specialised healthcare which captures only the more severe forms of morbidity. Due to different barriers to access healthcare that are specific to immigrants[36], we may have estimated differentially lower specialised healthcare use among refugees and non-refugee migrants than the Swedish-born. This may lead to misclassification of undiagnosed and/or untreated individuals as unexposed. Potential differences in healthcare-seeking behaviour in Swedish-born individuals and migrants may also affect our findings. Moreover, there was a lack of data on important covariates for exemple social connectedness and health behaviour. Furthermore, the rare outcome may lead to lower statistical power for some of the analyses. Additionally, generalisability of our results is limited to countries with similar healthcare and social welfare systems, and where the ethnic composition of the immigrant population is not significantly different than our study population. Moreover, these results can only be generalised to resettled refugees and non-refugee migrants dwelling in the community rather than those living in refugee camps or immigration detention centres.

Conclusions

The risk of all-cause mortality as well as specific mortality due to suicide and death due to external causes is lower among young refugees and non-refugee migrants compared to Swedish-born individuals. The lower rate of mortality of refugees can also be seen among those with specific mental and somatic disorders. With few exceptions, young migrants with specific mental and somatic disorders have a mortality advantage compared to their Swedish-born peers with the same disorders. Further research on protective factors is warranted.

For peer review only - http://bmjopen.bmj.com/site/about/guidelines.xhtml

Funding

The work was supported by the Swedish Research Council (grant number 2018-05783).

Conflict of interest

None declared.

Ethics approval

Ethical approval of this study was given by the Regional Ethical Board in Stockholm (2007/762-31). The Ethical board also waived the requirement of informed consent from the participants in this study.

Availability of data and material

These data cannot be made publicly available due to privacy regulations. According to the General Data Protection Regulation, the Swedish law SFS 2018:218, the Swedish Data Protection Act, the Swedish Ethical Review Act, and the Public Access to Information and Secrecy Act, these types of sensitive data can only be made available for specific purposes, including research, that meets the criteria for access to this type of sensitive and confidential data as determined by a legal review. Readers may contact Professor Kristina Alexanderson (kristina.alexanderson@ki.se) regarding the data.

It was not appropriate or possible to involve patients or the public in the design, or conduct, or reporting, or dissemination plans of our research.

Contributorship statement

The study was designed by MH, EB and EMR. EB conducted the data analyses. EMR and MH drafted the manuscript. SF, SR, AC, TD, KG and RA contributed with intellectual content and commented to results and successive drafts.

For peer review only

References

1. United Nations. International Migration Report: Highlights (ST/ESA/SER.A/404). New York: United Nations, Department of Economic and Social Affairs, Population Division, 2017.
2. UNHCR. Global trends: Forced displacement in 2017. In: Refugees UNHCf, ed. Global Trends, 2018:76.
3. Swedish Migration Agency. Applications for asylum received 2000-2019 (https://www.migrationsverket.se/English/About-the-Migration-Agency/Statistics/Asylum.html). Stockholm, 2020.
4. Sarkadi A, Ådahl K, Stenvall E, et al. Teaching Recovery Techniques: evaluation of a group intervention for unaccompanied refugee minors with symptoms of PTSD in Sweden. *European Child & Adolescent Psychiatry* 2017 doi: 10.1007/s00787-017-1093-9
5. Hodes M, Vasquez MM, Anagnostopoulos D, et al. Refugees in Europe: national overviews from key countries with a special focus on child and adolescent mental health. *European Child & Adolescent Psychiatry* 2018;27(4):389-99. doi: 10.1007/s00787-017-1094-8
6. Björkenstam E, Helgesson M, Norredam M, et al. Differences in psychiatric care utilization between refugees, non-refugee migrants and Swedish-born youth. *Psychol Med* 2020:1-11. doi: 10.1017/s0033291720003190 [published Online First: 2020/09/12]
7. Fazel M, Reed RV, Panter-Brick C, et al. Mental health of displaced and refugee children resettled in high-income countries: risk and protective factors. *The Lancet* 2012;379(9812):266-82. doi: 10.1016/s0140-6736(11)60051-2
8. Fazel M. Psychological and psychosocial interventions for refugee children resettled in high-income countries. *Epidemiology and psychiatric sciences* 2018;27(2):117-23. doi: 10.1017/S2045796017000695 [published Online First: 2017/11/10]
9. Norredam M, Nellums L, Nielsen RS, et al. Incidence of psychiatric disorders among accompanied and unaccompanied asylum-seeking children in Denmark: a nation-wide register-based cohort study. *European Child & Adolescent Psychiatry* 2018;27(4):439-46. doi: 10.1007/s00787-018-1122-3
10. Kien C, Sommer I, Faustmann A, et al. Prevalence of mental disorders in young refugees and asylum seekers in European Countries: a systematic review. *European Child & Adolescent Psychiatry* 2019;28(10):1295-310. doi: 10.1007/s00787-018-1215-z
11. Montgomery E. Trauma, exile and mental health in young refugees. *Acta psychiatrica Scandinavica* 2011;124(s440):1-46. doi: https://doi.org/10.1111/j.1600-0447.2011.01740.x
12. Manhica H, Almquist Y, Rostila M, et al. The use of psychiatric services by young adults who came to Sweden as teenage refugees: a national cohort study. *Epidemiology and psychiatric sciences* 2017;26(5):526-34. doi: 10.1017/s2045796016000445 [published Online First: 2016/06/30]
13. Patton GC, Coffey C, Sawyer SM, et al. Global patterns of mortality in young people: a systematic analysis of population health data. *Lancet* 2009;374(9693):881-92. doi: 10.1016/s0140-6736(09)60741-8 [published Online First: 2009/09/15]
14. Amin R, Helgesson M, Runeson B, et al. Suicide attempt and suicide in refugees in Sweden - a nationwide population-based cohort study. *Psychol Med* 2019:1-10. doi: 10.1017/s0033291719003167 [published Online First: 2019/12/21]
15. Björkenstam E, Helgesson M, Amin R, et al. Mental disorders, suicide attempt and suicide: differences in the association in refugees compared with Swedish-born individuals. *Br J Psychiatry* 2019:1-7. doi: 10.1192/bjp.2019.215 [published Online First: 2019/10/15]
16. Aldridge RW, Nellums LB, Bartlett S, et al. Global patterns of mortality in international migrants: a systematic review and meta-analysis. *Lancet* 2018;392(10164):2553-66. doi: 10.1016/s0140-6736(18)32781-8 [published Online First: 2018/12/12]
17. Helgesson M, Rahman S, Saboonchi F, et al. Disability pension and mortality in individuals with specific somatic and mental disorders: examining differences between refugees and

For peer review only - http://bmjopen.bmj.com/site/about/guidelines.xhtml

Swedish-born individuals. *J Epidemiol Community Health* 2021 doi: 10.1136/jech-2019-213436 [published Online First: 2021/01/22]
18. Guillot M, Khlat M, Elo I, et al. Understanding age variations in the migrant mortality advantage: An international comparative perspective. *PLoS One* 2018;13(6):e0199669. doi: 10.1371/journal.pone.0199669 [published Online First: 2018/06/30]
19. Helgesson M, Johansson B, Nordquist T, et al. Healthy migrant effect in the Swedish context: a register-based, longitudinal cohort study. *BMJ Open* 2019;9(3):e026972. doi: 10.1136/bmjopen-2018-026972 [published Online First: 2019/03/18]
20. Mittendorfer-Rutz E, Rasmussen F, Wasserman D. Familial clustering of suicidal behaviour and psychopathology in young suicide attempters. A register-based nested case control study. *Soc Psychiatry Psychiatr Epidemiol* 2008;43(1):28-36. doi: 10.1007/s00127-007-0266-0 [published Online First: 2007/10/16]
21. Hällgren J, Ösby U, Westman J, et al. Mortality trends in external causes of death in people with mental health disorders in Sweden, 1987-2010. *Scand J Public Health* 2019;47(2):121-26. doi: 10.1177/1403494818758912 [published Online First: 2018/03/02]
22. Björkenstam E, Helgesson M, Norredam M, et al. Common mental disorders among young refugees in Sweden: The role of education and duration of residency. *J Affect Disord* 2020;266:563-71. doi: 10.1016/j.jad.2020.02.015 [published Online First: 2020/02/15]
23. Hjern A. Migration and public health: Health in Sweden: The National Public Health Report 2012. Chapter 13. *Scand J Public Health* 2012;40(9 Suppl):255-67. doi: 10.1177/1403494812459610 [published Online First: 2012/12/19]
24. Kohlenberger J, Buber-Ennser I, Rengs B, et al. Barriers to health care access and service utilization of refugees in Austria: Evidence from a cross-sectional survey. *Health Policy* 2019;123(9):833-39. doi: 10.1016/j.healthpol.2019.01.014 [published Online First: 2019/03/18]
25. Hollander AC. Social inequalities in mental health and mortality among refugees and other immigrants to Sweden--epidemiological studies of register data. *Glob Health Action* 2013;6:21059. doi: 10.3402/gha.v6i0.21059 [published Online First: 2013/07/03]
26. Diaz E, Kumar BN, Gimeno-Feliu LA, et al. Multimorbidity among registered immigrants in Norway: the role of reason for migration and length of stay. *Trop Med Int Health* 2015;20(12):1805-14. doi: 10.1111/tmi.12615 [published Online First: 2015/10/02]
27. Thordardottir EB, Yin L, Hauksdottir A, et al. Mortality and major disease risk among migrants of the 1991-2001 Balkan wars to Sweden: A register-based cohort study. *PLoS medicine* 2020;17(12):e1003392. doi: 10.1371/journal.pmed.1003392 [published Online First: 2020/12/02]
28. Patrick D, Gadeyne S. Adult Migrant Mortality Advantage in Belgium: Evidence Using Census and Register Data. *Population (English Edition, 2002-)* 2005;60(5/6):655-98. doi: 10.2307/4148188
29. Gilliver SC, Sundquist J, Li X, et al. Recent research on the mental health of immigrants to Sweden: a literature review. *Eur J Public Health* 2014;24 Suppl 1:72-9. doi: 10.1093/eurpub/cku101 [published Online First: 2014/08/12]
30. Bas-Sarmiento P, Saucedo-Moreno MJ, Fernandez-Gutierrez M, et al. Mental Health in Immigrants Versus Native Population: A Systematic Review of the Literature. *Archives of psychiatric nursing* 2017;31(1):111-21. doi: 10.1016/j.apnu.2016.07.014 [published Online First: 2017/01/21]
31. Mittendorfer-Rutz E, Hagström A, Hollander AC. High Suicide Rates Among Unaccompanied Minors/Youth Seeking Asylum in Sweden. *Crisis* 2020;41(4):314-17. doi: 10.1027/0227-5910/a000636 [published Online First: 2019/12/21]
32. Amin R, Rahman S, Dorner TE, et al. Country of birth, time period of resettlement and subsequent treated common mental disorders in young refugees in Sweden. *Eur J Public Health* 2020 doi: 10.1093/eurpub/ckaa140 [published Online First: 2020/08/26]

For peer review only - http://bmjopen.bmj.com/site/about/guidelines.xhtml

33. Hvidtfeldt C, Petersen JH, Norredam M. Prolonged periods of waiting for an asylum decision and the risk of psychiatric diagnoses: a 22-year longitudinal cohort study from Denmark. *Int J Epidemiol* 2020;49(2):400-09. doi: 10.1093/ije/dyz091 [published Online First: 2019/05/21]
34. Hawton K, van Heeringen K. Suicide. *Lancet* 2009;373(9672):1372-81. doi: 10.1016/s0140-6736(09)60372-x [published Online First: 2009/04/21]
35. Graetz V, Rechel B, Groot W, et al. Utilization of health care services by migrants in Europe-a systematic literature review. *Br Med Bull* 2017;121(1):5-18. doi: 10.1093/bmb/ldw057 [published Online First: 2017/01/22]
36. Amin R, Rahman S, Tinghög P, et al. Healthcare use before and after suicide attempt in refugees and Swedish-born individuals. *Social psychiatry and psychiatric epidemiology* 2021;56:325-38. doi: 10.1007/s00127-020-01902-z

For peer review only

For peer review only - http://bmjopen.bmj.com/site/about/guidelines.xhtml

Supplementary file showing the directed acyclic graphs (DAG)

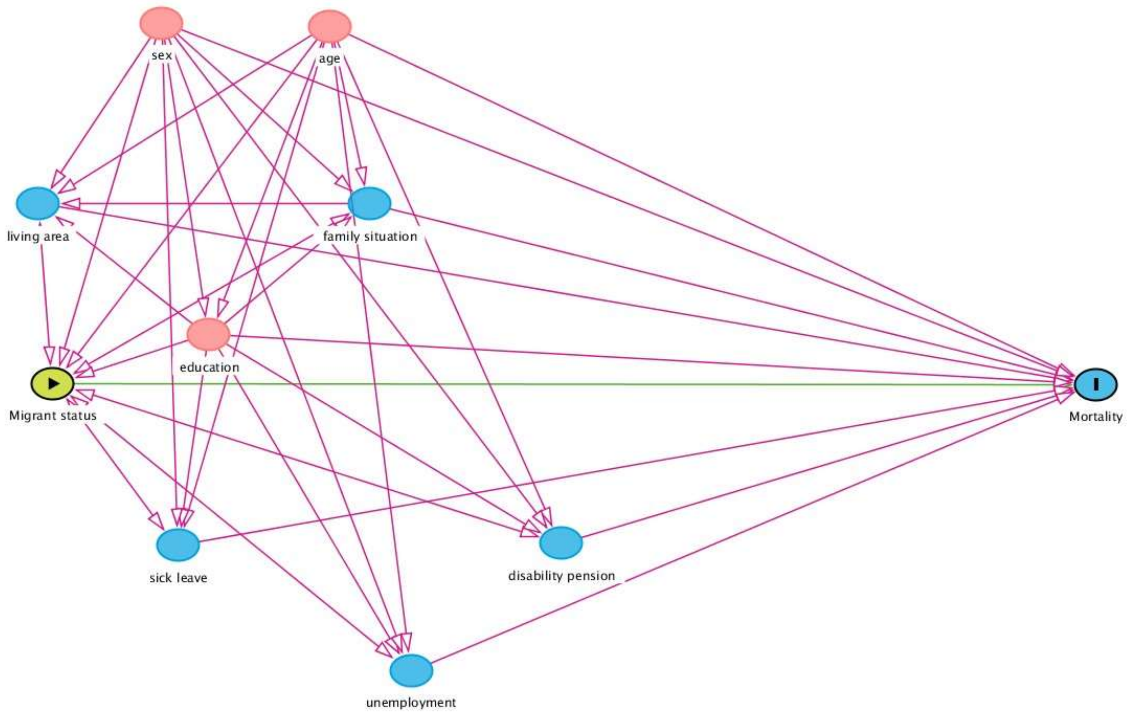

DAG for exposure (migrant status) and outcome (mortality) **unadjusted** for sociodemographic and Labour market marginalisation factors

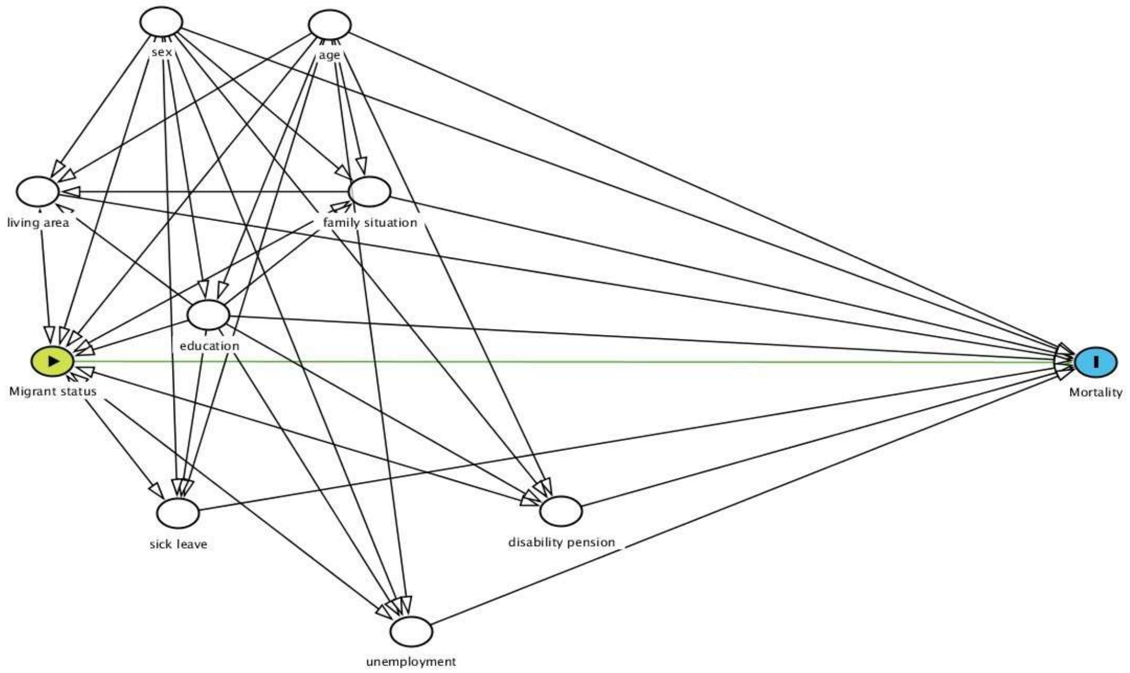

DAG for exposure (migrant status) and outcome (mortality) **adjusted** for **1)** sociodemographic and **2)** labour market marginalisation (LMM) factors

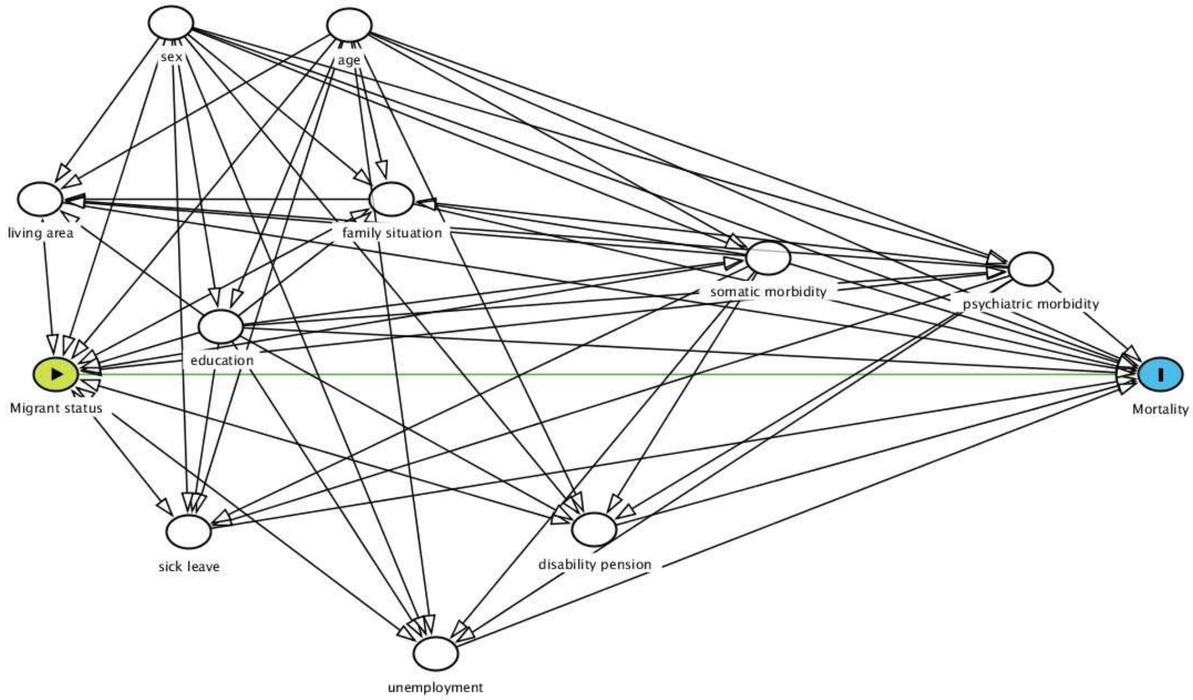

DAG for exposure (migrant status) and outcome (mortality) **adjusted** for **1)** sociodemographic, **2)** LMM and **3)** morbidity factors
